# Graph-Based Deep Learning for Medical Diagnosis and Analysis: Past, Present and Future

**DOI:** 10.3390/s21144758

**Published:** 2021-07-12

**Authors:** David Ahmedt-Aristizabal, Mohammad Ali Armin, Simon Denman, Clinton Fookes, Lars Petersson

**Affiliations:** 1 Imaging and Computer Vision Group, CSIRO Data61, Canberra 2601, Australia; ali.armin@data61.csiro.au (M.A.A.); lars.petersson@data61.csiro.au (L.P.); 2 Signal Processing, Artificial Intelligence and Vision Technologies (SAIVT) Research Program, Queensland University of Technology, Brisbane 4000, Australia; s.denman@qut.edu.au (S.D.); c.fookes@qut.edu.au (C.F.)

**Keywords:** graph representation, graph convolutional networks, brain functional connectivity, anatomical structure analysis

## Abstract

With the advances of data-driven machine learning research, a wide variety of prediction problems have been tackled. It has become critical to explore how machine learning and specifically deep learning methods can be exploited to analyse healthcare data. A major limitation of existing methods has been the focus on grid-like data; however, the structure of physiological recordings are often irregular and unordered, which makes it difficult to conceptualise them as a matrix. As such, graph neural networks have attracted significant attention by exploiting implicit information that resides in a biological system, with interacting nodes connected by edges whose weights can be determined by either temporal associations or anatomical junctions. In this survey, we thoroughly review the different types of graph architectures and their applications in healthcare. We provide an overview of these methods in a systematic manner, organized by their domain of application including functional connectivity, anatomical structure, and electrical-based analysis. We also outline the limitations of existing techniques and discuss potential directions for future research.

## 1. Introduction

Medical diagnosis refers to the process by which one can determine which disease or condition explains a patient’s symptoms. The required information for diagnosis is obtained from a patient’s medical history, and various medical tests that capture the patient’s functional and anatomical structures through diagnostic imaging methods such as functional magnetic resonance imaging (*f*MRI), magnetic resonance imaging (MRI), computed tomography (CT), and other diagnostic tools including the electroenchephalogram (EEG). However, given the often time-consuming diagnosis process which is prone to subjective interpretation and inter-observer variability, clinical experts have begun to benefit from computer-assisted interventions. Automation is of benefit in situations where there is limited access to healthcare services and physicians. Automation is also being pursued to increase the quality and decrease the cost of healthcare systems [1].

**Figure 1:**
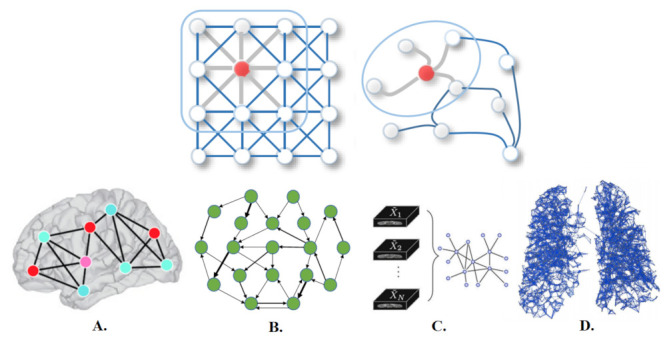
Traditional 2D grid representation and graph-based representation (the neighbours of a node are unordered and variable in size). (**A**,**B**) Brain graph of *f* MRI and EEG data for brain responses and emotion analysis, respectively; (**C**) DMRI sampling represented by a graph (DMRI brain reconstruction); (**D**) Graph-like representation for organ segmentation (CT -pulmonary airway). Image adapted from [4,5,6,7,8].

Deep learning offers an exciting avenue to address these demands. The success of deep learning in many fields is due in part to the availability of rapidly increasing computing resources and large experimental datasets, and in part to the ability of deep learning to extract representations from data structured as regular grids (i.e., images) through stacked convolutional operations. There are several review papers available that analyse the benefits of traditional machine learning and deep learning methods for the detection and segmentation of medical anomalies and anatomical structures, and computer-aided diagnosis [2,3]. Although CNNs have shown impressive performance in the medical field for imaging (MRI, CT) and non-imaging applications (*f*MRI, EEG), their conventional formulation is limited to data structured in an ordered, grid-like fashion. Several physical human processes generate data that are naturally embedded in a graph structure as illustrated in Figure 1 (Top). Traditional CNNs analyse local areas based on fixed connectivity (determined by the convolutional kernel), leading to limited performance, difficulty in interpreting the functional and anatomical structures being modeled, and an inability to capture complex neighbourhood information. Therefore, machine-learning models that can exploit graph structures are at an advantage as they enable an effective representation of complex physical entities and processes, and irregular relationships.

Graph networks belong to an emerging area that has also made a tremendous impact across many technological domains. Much of the information coming from disciplines such as chemistry, biology, genetics, and healthcare is not well suited to vector-based representations, and instead requires complex data structures. Graphs inherently capture relationships between entities, and are thus potentially very useful for these applications to encode relational information between variables [4]. Hence, effort has been devoted to the generalization of graph neural networks (GNN) into non-structural (unordered) and structural (ordered) scenarios. However, while the use of graph-based representations is becoming more common in the medical domain, such approaches are still scarce compared to conventional deep learning methods, and their potential to address many challenging medical problems is yet to be fully realised.

The adaptation of deep learning from images to graphs has resulted in a new cross-domain field of graph-based deep learning that seeks to learn informative representations of graphs in an end-to-end manner. Graph convolutional networks (GCNs) have extended the theory of signal processing on graphs [9] to enable the representation learning power of CNNs to be applied to irregular graph data. GCNs generalize the convolution operation to non-Euclidean graph data. The graph convolutional operation aims to generate representations for vertices by aggregating the features of a given vertex with the features of its neighbours. The relationship-aware representations generated by GCNs greatly enhance the discriminative power of CNN features, and the improved model interpretability can help clinicians to determine, for example, the parts of the brain that are most involved in one particular task. The popularity of the rapidly growing field of deep learning on GNNs is also reflected by the numerous recent surveys on graph representations and their applications. Existing reviews provide a comprehensive overview of deep learning for non-Euclidean data, graph deep learning frameworks and a taxonomy of existing techniques [4,10] or introduce general applications that cover biology and signal processing domains [11,12,13].

In this paper, we endeavour to provide a thorough and methodological review of multiple GNN models proposed for use in medical diagnosis and analysis. We seek to explain the fundamental reasons why GNNs are worth investigating for this domain, and highlight the emerging medical analytics challenges that GNNs are well placed to address. Although some papers have surveyed medical image analysis using deep learning techniques and have introduced the concept of GNNs for the assessment of neurological disorders [14], to the best of our knowledge, no systematic review exists that introduces and discusses the current applications of GNNs to unstructured medical data.

### 1.1. Why Graph-Based Deep Learning for Medical Diagnosis and Analysis?

Recent progress in deep learning has increased the potential of medical image analysis by enabling the discovery of morphological, textural, and temporal representations from images and signals solely from the data. GNNs have seen a surge in popularity due to their successes in modeling unstructured and structured relational data including brain signals (*f*MRI and EEG), and in the detection and segmentation of organs (MRI, CT) as represented in Figure 1 (Bottom). Below, we outline several application domains which are well suited to graph networks, and outline the reasons why graph neural networks are becoming more widely used within these domains.

#### 1.1.1. Brain Activity Analysis

Brain signals are an example of a graph signal, and the graph representation can encode the complex structure of the brain to represent either physical or functional connectivity across different brain regions. At the structural level, the network is defined by the anatomical connections between regions of brain tissue. At the functional level, the graph nodes represent brain regions of interest (ROI), while edges capture the relationships between the regions and their activities, computed via an *f*MRI correlation matrix [15].

GNN models also offer advantages when considering the need to develop deep-learning models that allow a direct interpretation of non-Euclidean spaces. The explanations obtained by such models can help to identify and localize regions relevant to a model’s decisions for a given task. An example is how certain brain regions, defined as biomarkers, are related to a specific neurological disorder [16,17].

Graphs also provide a natural way to represent population data and model complex interactions and associations between subjects for disease analysis [18].

#### 1.1.2. Brain Surface Representation

The structures in medical images have a spherical topology (i.e., brain cortical or subcortical surfaces). These are often represented by triangular meshes with large inter- and intra-subject variations in vertex numbers and changes in local connectivity. Due to the absence of a consistent and regular neighbourhood definition, conventional CNNs cannot be directly applied to these surfaces [19]. GCNs, however, can be applied to graphs with a varying number of nodes and connectivity [20]. Spherical CNN architectures can render valid parametrizations in a spherical space without introducing spatial distortions on the sphere (spherical mapping) [21], and geometric features can be augmented by utilizing surface registration methods [22]. GCNs can also offer more flexibility to parcellate the cerebral cortex (surface segmentation) by providing better generalization on target-domain datasets where surface data are aligned differently, without the need for manual annotations or explicit alignment of these surfaces [23].

#### 1.1.3. Segmentation and Labeling of Anatomical Structures

Segmentation of vessels and organs is a critical but challenging stage in the medical image processing pipeline due to anatomical complexity. Traditional deep learning segmentation approaches classify each pixel of an image into a class by extracting high-level semantic features. CNNs struggle because regions in images are rarely grid-like and require non-local information. Compared with these pixel-wise methods, a graph-based method learns and regresses the location of the vessels and organs directly, and allows the model to learn local spatial structures [24,25]. GCNs can also propagate and exchange local information across the whole image to learn the semantic relationships between objects.

### 1.2. Scope of Review

The application of graph neural networks to medical signal processing and analysis is still in its nascent stages. In this paper, we present a survey that captures the current efforts to apply GNNs for medical data understanding and diagnosis The total number of applications considered in our survey is 92 with a chronology of publication as follows: 2017 (4), 2018 (7), 2019 (37), 2020 (40), and 2021 (4). The area of digital pathology (WSI) is omitted from this review due to the diverse applications of GCNs to this domain, which we feel merit their own separate review paper [26].

### 1.3. Contribution and Organisation

Compared to other recent reviews that cover the theoretical aspects of graph networks in multiple domains, our manuscript has novel contributions which are summarized as follows:

1. We identify a number of challenges facing traditional deep learning when applied to medical signal analysis, and highlight the contributions of graph neural networks to overcome these.
2. We introduce and discuss diverse graph frameworks proposed for medical diagnosis and their specific applications. We cover work for biomedical imaging applications using graph networks combined with deep learning techniques.
3. We summarise the current challenges encountered by graph-based deep learning and propose future directions in healthcare based on currently observed trends and limitations.

In Section 2, we briefly describe the most common graph-based deep learning models used in this domain, including GCNs and its variants, with temporal dependencies and attention structures.

In Section 3, we explain the use cases identified in the literature review. We organise publications according to the input data (functional connectivity, electrical-based, and anatomical structure) and cluster approaches based on specific applications (e.g., Alzheimer’s disease, organ segmentation, or brain data regression).

Finally, Section 4 highlights the limitation of current GNNs adopted for medical diagnosis and introduces graph-based deep learning techniques that can be utilised in this domain. We also provide some research directions and future possibilities for the use of GNNs in healthcare that have not been covered in the literature, such as for behavioural analysis.

## 2. Graph Neural Networks Background

In this section, we introduce several graph-based deep learning models including GCNs and their variants with temporal dependencies, and attention structures, which have been used as the foundation for the medical applications. We aim to provide technical insights regarding the architectures. A deep analysis of each architecture can be found in multiple survey papers in this domain [4,12,13].

### 2.1. Graph Representation

**Figure 2:**
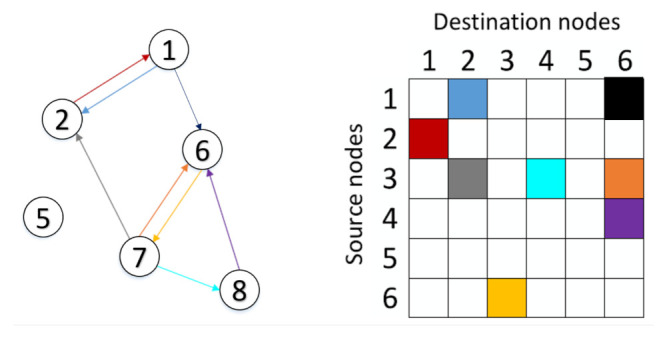
Example of a directed graph (**Left**) and the corresponding adjacency matrix (**Right**). Image adapted from [6].

A graph can be represented as G=(V,E,W), where *V* represents the set of *N* nodes, |V|=N; E denotes the set of edges connecting these nodes, and *W* is the adjacency matrix. The adjacency matrix describes the connections between any two nodes in V, in which the importance of the connection between the *i*-th and the *j*-th nodes is measured by the entry of *W* in the *i*-th row and *j*-th column, and denoted by $w_{ij}$. Commonly used methods to determine the entries, $w_{ij}$, of *W* include the Pearson correlation-based graph, the K-nearest neighbour (KNN) rule method, and the distance-based graph [9]. Figure 2 demonstrates an example of a graph containing six vertices and the edges connecting the nodes of the graph, along with the graph adjacency matrix.

### 2.2. Graph Neural Network Architectures

Graph convolutional networks learn abstract feature representations for each feature in a node via message passing, in which nodes iteratively aggregate feature vectors from their neighbourhood to compute a new feature vector at the next hidden layer in the network. Different GNN variants use different aggregators to gather information from each node’s neighbours, and use varied methods to update the hidden states of nodes.

GCNs can be categorised as: spectral-based [27,28] and spatial-based [29,30]. Spectral-based GCNs rely on the concept of spectral convolutional neural networks that build upon the graph Fourier transform and the normalized Laplacian matrix of the graph. Spatial-based GCNs define a graph convolution operation based on the spatial relationships that exist among the graph nodes.

Based on the original graph neural networks in [31], we explore the most representative GNN variants that have been proposed for several clinical applications.

#### 2.2.1. ChebNet

For spectral-based GCNs, the convolution operation is defined in the Fourier domain by computing the eigendecomposition of the graph Laplacian [32]. The normalized graph Laplacian is defined as $L=I_{N}-D^{-1/2}AD^{-1/2}=U\Lambda U^{T}$ (*D* is the degree matrix and *A* is the adjacency matrix of the graph), where the columns of *U* is the matrix of eigenvectors and Λ is a diagonal matrix of its eigenvalues. The operation can be defined as the multiplication of a signal $x\in R^{N}$ (a scalar for each node) with a filter $g_{\theta }=\mathrm{diag}\left(\theta \right)$, parameterized by $\theta \in R^{N}$

(1) $$g_{\theta }🟉x=Ug_{\theta }\left(\Lambda \right)U^{T}x.$$

Defferrard et al. [27] proposed the ChebyNet, which approximates the spectral filters by truncated Chebyshev polynomials, avoiding the computation of the Fourier basis. A Chebyshev polynomial $T_{m}\left(x\right)$ of order *m* evaluated at $\tilde{L}$ is used [27], and the operation is defined as

(2) $$g_{\theta }🟉x\approx \sum_{m=0}^{M-1}\theta_{m}T_{m}\left(\tilde{L}\right)x,$$

where $\tilde{L}$ is a diagonal matrix of scaled eigenvalues defined as $\tilde{L}=2L\;/\;\lambda_{\max}-I_{N}$. $\lambda_{\max}$ denotes the largest eigenvalue of *L*. The Chebyshev polynomials are defined as $T_{m}\left(x\right)=2xT_{k-1}\left(x\right)-T_{k-2}\left(x\right)$ with $T_{0}\left(x\right)=1$ and $T_{1}\left(x\right)=x$. By introducing Chebyshev polynomials, ChebNet does not require calculating the eigenvectors of the Laplacian matrix, and this reduces the computational cost. A graph pooling layer in the GCN pools information from multiple vertices to one vertex, which reduces the graph size and expands the receptive field of the graph filters. The feature vectors from the last graph convolutional layer are concatenated into a single feature vector, which is fed to a fully connected layer to obtain classification results.

#### 2.2.2. Graph Convolutional Network

A GCN is a spectral-GNN with mean pooling aggregation. Kipf and Welling [28] presented the GCN using a localized first-order approximation of spectral convolutions on the graph. It uses a simple layer-wise propagation rule to encode the relationships of nodes from the graph structure into node features. By reducing the size of the convolution filter K=1 to alleviate the problem of overfitting to the local neighbourhood structure of graphs with a very wide node degree distribution [28], and a further approximation λ≈2, Equation (2) can be simplified to

(3) $$g_{\theta }🟉x\approx \theta_{0}^{{}^{\prime }}x+\theta_{1}^{{}^{\prime }}x\left(L-I_{N}\right)x=\theta_{0}^{{}^{\prime }}x+\theta_{1}^{{}^{\prime }}D^{-1/2}AD^{-1/2}x.$$

Here, $\theta_{0}^{{}^{\prime }},\theta_{1}^{{}^{\prime }}$ are two unconstrained variables. To restrain the number of parameters and avoid overfitting, GCN further assume that $\theta =\theta_{0}^{{}^{\prime }}=-\theta_{1}^{{}^{\prime }}$, leading to the following definition of a graph convolution:

(4) $$g_{\theta }🟉x\approx \theta \left(I_{N}+D^{-1/2}AD^{-1/2}\right)x.$$

Stacking this operation will cause numerical instabilities and the explosion or disappearance of gradients. Thus, Kipf and Welling [28] generalize the definition to a signal $X\in R^{NXC}$ with *C* input channels and *F* filters for feature maps as follows:

(5) $$Z={\tilde{D}}^{-1/2}\tilde{A}{\tilde{D}}^{-1/2}X\Theta ,$$

where $\Theta \in R^{CXF}$ is the matrix formed by the filter bank parameters, and $Z\in R^{NXF}$ is the signal matrix obtained by convolution.

#### 2.2.3. GraphSAGE

GraphSAGE is a spatial-GCN which uses a node embedding with max-pooling aggregation. Hamilton et al. [30] offer an extension of using GCNs for inductive unsupervised representation learning with trainable aggregation functions instead of simple convolutions applied to neighbourhoods in a GCN. The *AGGREGATE* operation can aggregate neighbouring node representations of the center node, while the *COMBINE* operation combines the neighbourhood node representation with the center node representation to obtain the updated center node representation. The authors propose a batch-training algorithm for GCNs to save memory at the cost of sacrificing time efficiency. The GraphSAGE framework generates embeddings by sampling and aggregating features from a node’s local neighbourhood,

(6) $$\begin{array}{c} h_{N_{v}}^{t}={\mathrm{AGGREGATE}}_{t}\left(\left\{h_{u}^{t-1},\forall u\in N_{v}\right\}\right),\\ h_{v}^{t}=\sigma \left(W^{t}\cdot \left[h_{v}^{t-1}∥h_{N_{v}}^{t}\right]\right), \end{array}$$

where $N_{v}$ is the neighbourhood set of node *v*, $h_{v}^{t}$ is the hidden state of node *v* at time step *t*, and $W^{t}$ is the weight matrix at layer *t*. Finally, σ denotes the logistic sigmoid function and ∥ denotes vector concatenation.

In [30], three aggregating functions are proposed: the element-wise mean, an LSTM, and max-pooling. The mean aggregator is an approximation of the convolutional operation from the transductive GCN framework [28]. An LSTM is adapted to operate on an unordered set by permutating the node’s neighbours. In the pooling aggregator, each neighbour’s hidden state is fed through a fully-connected layer, and then a max-pooling operation is applied to the set of the node’s neighbours. Unlike GCN’s aggregator, which assigns neighbour-specific, predefined weights based on node degree, GraphSAGE’s mean operator assigns the same weights to all neighbours of a given node.

#### 2.2.4. Graph Isomorphism Network

The graph isomorphism network (GIN) [33] is a spatial-GCN that aggregates neighbourhood information by summing the representations of neighbouring nodes. Isomorphism graph-based models are designed to interpret graphs with different nodes and edges. GIN’s aggregation and readout functions are injective and thus are designed to achieve maximum discriminative power [33].

#### 2.2.5. Graph Networks with Attention Mechanisms

Attention mechanisms are established in neuroscience and can be divided into two main types: soft-attention and self-attention mechanisms.

*Soft-attention mechanisms:* Soft-attention mechanisms allow the model to learn the most relevant parts of the input sequence during training. Soft-attention mechanisms are end-to-end approaches that can be learned by gradient-based methods [34]. Attention also provides a tool for interpreting network results and discovering the underlying dependencies that have been learnt. The attention mechanism can be formulated as follows:

(7) $$\begin{array}{c} u_{t}=\tanh\left(Wh_{t}+b\right),\\ \alpha_{t}=\frac{\exp(u_{t}^{T}u_{w})}{\sum_{j=1}^{n}\exp\left(u_{t}^{T}u_{w}\right)},\\ s_{t}=\sum_{t}\alpha_{t}h_{t}, \end{array}$$

where $h_{t}$ is the output of each layer; *W*, $u_{w}$, and *b* are trainable weights and bias. The importance of each element in $h_{t}$ is measured by estimating the similarity between $u_{t}$ and $h_{t}$, which is randomly initialized. $\alpha_{t}$ is a softmax function. The scores are multiplied by the hidden states to calculate the weighted combination, $s_{t}$ (attention-based final output).

*Self-attention mechanisms* Graph attention networks (GAT) [35] incorporate the attention mechanism into the propagation steps by modifying the convolution operation. In a traditional GCN, the weights typically depend on the degree of the neighbouring nodes, while, in GATs, the weights are computed by a self-attention mechanism based on node features (i.e., to learn neighbour-specific weights). Veličković et al. [35] constructed a graph attention network by stacking a single graph attention layer, *a*, which is a single-layer feedforward neural network, parametrized by a weight vector $\vec{a}\in R^{2F^{i}}$. The layer computes the coefficients in the attention mechanisms of the node pair (i,j) by

(8) $$\alpha_{i,j}=\frac{\exp(\mathrm{LeakyReLu}\left({\vec{a}}^{T}\left[W{\vec{h}}_{i}∥W{\vec{h}}_{j}\right]\right))}{\sum_{k\in N_{i}N}\exp\left(\mathrm{LeakyReLu}\left({\vec{a}}^{T}\left[W{\vec{h}}_{i}∥W{\vec{h}}_{k}\right]\right)\right)},$$

where ∥ represents the concatenation operation. The attention layer takes as input a set of node features $h=\left\{\vec{h_{1}},\vec{h_{2}},...,\vec{h_{N}}\right\},\vec{h_{i}}\in R^{F}$, where *N* is the number of nodes of the input graph and *F* the number of features for each node, and produces a new set of node features $h^{{}^{\prime }}=\left\{{\vec{h_{1}}}^{{}^{\prime }},{\vec{h_{2}}}^{{}^{\prime }},...,{\vec{h_{N}}}^{{}^{\prime }}\right\},{\vec{h_{i}}}^{{}^{\prime }}\in R^{F}$ as its output. To generate higher-level features, as an initial step, a shared linear transformation, parametrized by a weight matrix $W\in R^{F^{\prime }*F}$, is applied to every node and subsequently a masked attention mechanism can be applied to every node, resulting in the following scores:

(9) $$e_{ij}=a\left(W\vec{h_{i}},W\vec{h_{j}}\right),$$

which indicates the importance of node $j^{{}^{\prime }}s$ features to node *i*. The final output feature of each node can be obtained by applying a nonlinearity, σ,

(10) $$h_{i}^{{}^{\prime }}=\sigma \left(\sum_{j\in N_{i}}\alpha_{ij}Wh_{j}\right).$$

The layer also uses multi-head attention to stabilise the learning process. *K* different attention heads are applied to compute mutually independent features in parallel, and then concatenate their features, resulting in the following representations:

(11) $$h_{i}^{{}^{\prime }}{=∥}_{K=1}^{K}\sigma \left(\sum_{j\in N_{i}}\alpha_{ij}^{k}W^{k}\vec{h_{j}}\right),$$

or by employing averaging and delay applying the final nonlinearity (usually a softmax or logistic sigmoid for classification problems),

(12) $$h_{i}^{{}^{\prime }}=\sigma \left(\frac{1}{K}\sum_{k=1}^{K}\sum_{j\in N_{i}}\alpha_{ij}^{k}W^{k}\vec{h_{j}}\right),$$

where $\alpha_{ij}^{k}$ is the normalized attention coefficient computed by the *k*-th attention mechanism.

Other GNN variants that were proposed in the paper surveyed in this review can be summarized as:

- Adaptive graph convolutional network [36].
- Graph domain adaptation [23].
- Isomorphism graph-based model [16].
- Synergic GCN [37,38].
- Simple graph convolution network [39,40].
- Graph-based segmentation (e.g., 3D Unet-graph [24,41], Spherical Unet [19,22]).
- Attention mechanisms for feature representation [42].
- Weighted GATs [43].
- Edge-weighted GATs [17,44].
- Attention based ST-GCN [45,46].
- Cross-modality with GAT-based embedding [47].

### 2.3. Graph Neural Networks with Temporal Dependency

GNNs have primarily been developed for static graphs that do not change over time. However, several real-world graphs are dynamic and evolve over time (e.g., brain activity recorded using *f*MRI). These variants of GNNs known as dynamic graphs aim to learn hidden patterns from the spatial and temporal dependencies of a graph. These models can be divided into two main types:

- RNN-based approaches: These methods capture spatio-temporal dependencies by using graph convolutions to filter inputs and hidden states before passing them to a recurrent unit.
- CNN-based approaches: These approaches tackle spatio–temporal graphs in a non-recursive manner. They use temporal connections to extend static graph structures so that they can apply traditional GNNs on the extended graphs.

#### 2.3.1. RNN-Based Approaches

The aim of these models is to learn node representations with recurrent neural architectures (RNNs). They assume a node in a graph constantly exchanges information/messages with its neighbours until a stable equilibrium is reached. In a deep learning model, RNNs introduce the notion of time by including recurrent edges that span adjacent time steps [48]. RNNs perform the same task for every element of a sequence, with the output being dependent on the previous computations and is therefore termed recurrent. LSTMs [49] were proposed to increase the flexibility of RNNs by employing an internal memory, termed the cell state, to address the vanishing gradient problem. Three logic gates are also introduced to adjust the cell state and produce the LSTM output. GRUs [50] are a variant of LSTMs which combines the forget and input gates to simplify the model.

*DCRNN model*: Diffusion convolutional recurrent neural networks (DCRNNs) [51] introduce the diffusion graph convolutional layer to capture spatial dependencies, and uses a sequence-to-sequence architecture with GRUs to capture temporal dependencies. A DCRNN uses a graph diffusion convolution layer to process the inputs of a GRU such that the recurrent unit receives historic information from the last time step as well as neighbourhood information from the graph convolution. The advantage of a DCRNN is its ability to handle long-term dependencies because of the recurrent network architectures.

*GCRN model*: The graph convolutional recurrent network (GCRN) [52] combines an LSTM network with ChebNet. A dynamic graph consists of time-varying connectivity among ROIs, and temporal information is handled by using LSTM units. Such a framework has been used in [53] for Alzheimer’s disease classification.

#### 2.3.2. CNN-Based Approaches

Although RNN-based models are widely used for time series analysis, they still suffer from time-consuming iterations, complex gate mechanisms, and slow response to dynamic changes. CNN-based approaches operate with fast training, stable gradients, and low memory requirements [54]. These approaches interleave 1D-CNN layers with graph convolutional layers to learn temporal and spatial dependencies, respectively.

*STGCN model*: The spatio-temporal graph convolutional network proposed by Yu et al. [55] employed convolutional structures on the time axis to capture dynamic temporal behaviors. This model integrates a 1D convolutional layer with ChebNet or GCN layers.

Such adoption of CNNs to perform a convolution operation in the temporal dimension has been used for sleep state classification [45].

*ST-GCN model*: ST-GCNs are popular for solving problems that base predictions on graph-structured time series [56]. The main benefits of a temporal GCN are that it uses a feature extraction operation that is shared over time and space. The input to the ST-GCN is the joint coordinate vectors on the graph nodes. Multiple layers of spatio-temporal graph convolution operations process the input data and higher-level feature maps on the graph. The resultant classification is performed using a conventional dense layer and activation.

*TGCN model*: Traditional temporal convolutional neural networks (TCNN) show that variations of convolutional neural networks can achieve impressive results for sequential data [57]. TCNNs use dilated causal convolutional layers where an output at time *t* is convolved only with elements from time *t* or earlier in the previous layer, i.e., inputs have no influence on output steps that precede them in time. In a dilated convolutional layer, a filter is sequentially applied to inputs by skipping input values with a pre-defined step (dilatation rate). Wu et al. [58] proposed a method for multi-resolution modeling of temporal dependencies; their temporal model is based on dilated convolutions. This approach is based on the fact that subsequent layers have dilated receptive fields. Temporal graph convolutional networks (TGCN) take structural times series data as input and apply feature extraction operations that are shared over both time and space. TGCNs show promise in applications such as EEG electrode distributions, where several datasets of similar but not identical configurations need to be analyzed.

Other dynamic GNN variants adopted and introduced by research analysed in this review include:

- Sequential GCN based on complex networks [59].
- Temporal-adaptive GCN [60].

## 3. Case Studies of GNN for Medical Diagnosis and Analysis

Graph convolutional networks have been utilized in classification, prediction, segmentation and reconstruction tasks with non-structural (e.g., *f*MRI, EEG) and structural data (e.g., MRI, CT). There are several specificities in the usage of GNNs in each of the medical signals identified by our survey that we review in the following sections.

The case studies for medical diagnosis are organised according to the input data and baseline graph framework adopted or proposed with its corresponding application and dataset. Case studies have been divided into four main groups: functional connectivity analysis, electrical-based analysis and anatomical structure analysis classification/regression and segmentation, which are detailed in Table 1, Table 2, Table 3 and Table 4, respectively. Rather than presenting an exhaustive literature review for each studied case, we discuss prominent highlights of how GNNs were used in each case.

It is important to highlight that there are several interesting works that aim to map functions to brain regions, to model the non-stationary nature of functional connectivity, and analyse the brain’s responses to internal or external events using graph-based deep learning models. These approaches have been used for gender classification with brain connectivity or brain structure, emotion recognition, and brain motor imagery. Although the outcome of these studies can be used for potentially clinical applications, they are not directly related to detecting or classifying a disease. Thus, their contributions are not covered in this manuscript.

### 3.1. Functional Connectivity Analysis

This section mainly covers application of graph learning representation on brain functional connectivity as summarized in Table 1; to the best of our knowledge, there are no applications that involved other body functions in the reviewed literature.

**Table 1:** Summary of GCN approaches adopted for functional connectivity and their applications.

Authors	Year	Modality	Application	Dataset
Li et al. [17] †	2020	t-*f*MRI	Classification: Autism disorder	ASD Biopoint Task (Yale Child Study Center [16]) (2 classes)
Li et al. [61]	2020	t-*f*MRI	Classification: Autism disorder	Biopoint [62] (2 classes)
Huang et al. [18]	2020	rs-*f*MRI	Classification: Autism disorder	ABIDE [63] (2 classes)
Rakhimberdina et al. [64]	2020	*f*MRI	Classification: Autism disorder	ABIDE [63] (2 classes)
Li et al. [65]	2020	t-*f*MRI	Classification: Autism disorder	Yale Child Study Center [16] (2 classes)
Jiang et al. [66]	2020	*f*MRI	Classification: Autism disorder	ABIDE [63] (2 classes)
Li et al. [16]	2019	t-*f*MRI	Classification: Autism disorder	Yale Child Study Center (private) (2 classes)
Kazi et al. [67]	2019	rs-*f*MRI	Classification: Autism disorder	ABIDE [63] (2 classes)
Yao et al. [68]	2019	rs-*f*MRI	Classification: Autism disorder	ABIDE [63] (2 classes)
Anirudh et al. [69]	2019	rs-*f*MRI	Classification: Autism disorder	ABIDE [63] (2 classes)
Rakhimberdina and Murata [40]	2019	*f*MRI	Classification: Autism disorder	ABIDE [63] (2 classes)
Ktena et al. [70]	2018	rs-*f*MRI	Classification: Autism disorder	ABIDE [63] (2 classes)
Parisot et al. [15]	2018	rs-*f*MRI	Classification: Autism disorder	ABIDE [63] (2 classes)
Ktena et al. [71]	2017	rs-*f*MRI	Classification: Autism disorder	ABIDE [63] (2 classes)
Parisot et al. [72]	2017	rs-*f*MRI	Classification: Autism disorder	ABIDE [63] (2 classes)
Rakhimberdina and Murata [40]	2019	*f*MRI	Classification: Schizophrenia	COBRE [73] (2 classes)
Rakhimberdina and Murata [40]	2019	rs-*f*MRI	Classification: Attention deficit disorder	ADHD-200 [74] (2 classes)
Yao et al. [68]	2019	rs-*f*MRI	Classification: Attention deficit disorder	ADHD-200 [74] (2 classes)
Yao et al. [60] 🟉	2020	rs-*f*MRI	Classification: Major depressive disorder	MDD [75] (2 classes)
Yang et al. [44] †	2019	*f*MRI/sMRI	Classification: Bipolar disorder	BD (private)
Li et al. [61]	2020	rs-*f*MRI	Classification: Brain response stimuli	HCP 900 [76] (7 classes)
Zhang et al. [5]	2019	*f*MRI	Classification: Brain response stimuli	HCP S1200 [76] (21 classes)
Guo et al. [77]	2017	MEG	Classification: Brain response stimuli	Visual stimulus (private) (2 classes)

🟉 GCN with temporal structures for medical diagnostic analysis. † GCN with attention structures for medical diagnostic analysis.

#### 3.1.1. Autism Spectrum Disorder

Autism spectrum disorder (ASD) is a complex neurodevelopmental disorder characterized by recurring difficulties in social interaction, speech and nonverbal communication, and restricted/repetitive behaviours. The screening of ASD is challenging due to uncertainties associated with its symptoms [78]. Resting-state *f*MRI (rs-*f*MRI) and task *f*MRI are the main modalities which are used to classify the population into ASD or health control (HC) groups.

**Figure 3:**
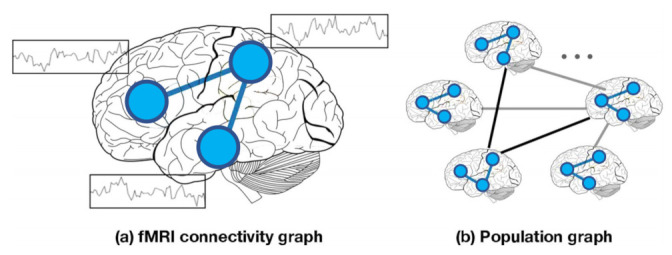
Proposed graph-based approaches for modeling with rs-*f*MRI data. Image taken from [40].

The rapid development of GNNs has attracted interest in using these architectures to analyse *f*MRI and non-imaging data for disease classification. Graph-based models can be classified into two groups based on the node definition as illustrated in Figure 3: (a) Individual graph: nodes are brain regions and edges are functional correlations between time series observations from those regions. Therefore, each graph represents only one subject and graph comparison metrics are computed to analyse these graphs, which are represented in the left panel in Figure 3; (b) Population graph: in this approach, each node represents a subject with corresponding brain-connectivity data, and edges are determined as the similarity between subjects’ phenotypic features (age, gender, handedness, etc.), as is shown in the right panel in Figure 3.

*Individual-based graph methods:* Ktena et al. [71] proposed a GNN method to learn a similarity (distance) metric between irregular graphs, such as the functional connectivity graphs obtained from the Autism Brain imaging Data Exchange (ABIDE) dataset [63], to classify individuals as autism spectrum disorder (ASD) or healthy controls (HC).

**Figure 4:**
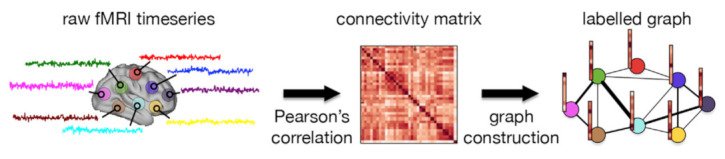
Estimation of single subject connectivity matrix and labelled graph representation. Pearson’s correlation coefficient is used to obtain a functional connectivity matrix from the raw *f*MRI time series. Image taken from [70] .

The method of Ktena et al. [70] is based on their previous work [71] to learn a graph similarity metric in spectral graph domain obtained from brain connectivity networks via supervised learning. They applied their method to individual graphs constructed from the ABIDE database to classify subjects into ASD or HC. The graph construction is illustrated in Figure 4. They showed that their spectral graph matching method not only outperforms non-graph matching but is also superior to individual subject classification and manifold learning methods.

The graph similarity metric proposed by Ktena et al. [70] using a specific template for brain region of interest (ROI) parcellation could impose a limitation such as analysis of single spatial scale (i.e., a fixed graph). Yao et al. [68] dealt with this limitation by proposing a multi-scale triplet GCN. They constructed multi-scale functional connectivity patterns for each subject through multi-scale templates for coarse-to-fine ROI parcellation. A triple GCN model was designed to learn multi-scale graph features of brain networks. Their application on *f*MRI data obtained from the ABIDE dataset showed their high performance in ASD and HC classification.

For GCN methods, all nodes are required to be presented during training which result in low performance on unseen nodes. Li et al. [16] proposed a GCN algorithm to discover ASD brain biomarkers from t-*f*MRI. Different from the semi-supervised spectral GCN algorithm [28] used in [72], this GCN classifier is isomorphism graph-based which can interpret graphs with different nodes and edges. In other words, the GCN is trained on the whole graph and tested on sub-graphs, such that they could determine the importance of sub-graphs and nodes. In both works from Li et al. [17,61], the authors also improved their individual graph level analysis by proposing a BrainGNN and a pooling regularized GNN model to investigate the brain region related to a neurological disorder from t-*f*MRI data for ASD or HC classification.

In addition, the low signal-to-noise ratio of *f*MRI and its high dimensionality impose another limitation on using *f*MRI for graph level classification and detection of functional differences between ASD and HC groups. Li et al. [65] dealt with this challenge by modeling the whole brain *f*MRI as a graph. This allowed them to preserve the geometrical and temporal information and learn a better graph embedding. They implemented their method on a group of 75 ASD children and 43 age- and IQ-matched healthy controls collected at the Yale Child Study Center [16]. Their results indicated a more robust classification of ASD or HC.

*Population-based graph methods:* Population graphs have been shown to be effective for brain disorder classification. Parisot et al. [72] investigated the performance of GCN for brain analysis in a population where the authors built a population graph using both rs-*f*MRI and non-imaging data (acquisition information). They applied their model on the ABIDE dataset [63] to classify subjects as ASD or HC. Their semi-supervised method showed better performance in comparison to a standard linear classifier (which only considered the individual features for classification). In an extension of this work, Parisot et al. [15] proposed a spectral GCN model which takes into account both the pairwise similarity between subjects (phenotypic information) and information obtained from subject-specific imaging features to classify subjects as ASD or HC in a population.

**Figure 5:**
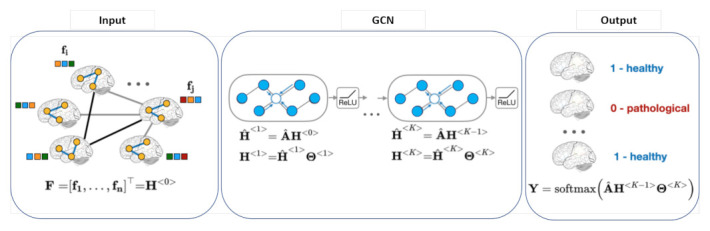
Proposed population graph-based approaches for subject classification. Image taken from [40].

As illustrated in Figure 5, Rakhimberdina and Murata [40] applied a linear simple graph convolution (SGC) [39] for brain disorder classification. They construct the population graphs by using the Hamming distance between phenotypic features of the subjects as weights of the edges of the graph. Their results on the ABIDE dataset [63] showed a high performance and efficiency of the linear SGC over the GCN based model deployed by Parisot et al. [15] on the same dataset.

As there is no standard method to construct graphs for a GNN, Anirudh et al. [69] proposed a bootstrapped version of GCNs that made models less sensitive to the initialisation of the construction of the population graph. They generated random graphs from the initial population graph (from the ABIDE dataset [63]) to train weakly a GCN for ASD and HC classification, and fused their prediction as the final result. To avoid the spatial limitation of a single template and learn multi-scale graph features of brain networks, Yao et al. [68] proposed a multi-scale triplet GCN model. These solutions, however, are problem specific, and choosing a particular graph definition over the other has remained a challenging problem. Rakhimberdina et al. [64] proposed a population graph-based multi-model ensemble method to deal with this problem. Their results on the ABIDE dataset [63] showed a 2.91% improvement in comparison to the best result reported for a non-graph solution [79].

The heterogeneity of the graph is challenging. Kazi et al. [67] proposed Inception-GCN as a spectral domain architecture for deep learning on graphs for node-level classification of disease prediction. This inception graph model is capable of capturing intra- and inter-graph structural heterogeneity during convolutions. The Inception-GCN could improve the performance of node classification in comparison to Parisot [72] as the baseline GCN using s-*f*MRI data from ABIDE.

To preserve the the topology information in the population network and their associated individual brain function network, Jiang et al. [66] proposed a hierarchical GCN framework to map the brain network to a low-dimensional vector while preserving the topology information. Their method leveraged a correlation mechanism in populating the network which could capture more information and result in more accurate brain network representation, and thus better classification of ASD from the ABIDE dataset [63] in comparison to Eigenpooling GCN [80] and the other population GCN [72] methods.

Finally, as stated earlier, uncertainties associated with ASD make it challenging [78], and thus Huang et al. [18] proposed an Edge-Variational GCN (EV-GCN) model with a learnable adaptive population graph core to incorporate multi-modal data for uncertainty-aware disease detection. Their model was tested on ASD/HC data, collected at the Yale Child Study Center [16] and showed the efficacy of the proposed method for embedding ASD and HC brain graphs.

#### 3.1.2. Schizophrenia

Automatic classification of schizophrenia (SZ) based on *f*MRI data has also attracted attention. SZ is a devastating mental disease with extraordinary complexity characterized by behavioral symptoms such as hallucinations and disorganized speech. SZ shows local abnormalities in brain activity and in functional connectivity networks which can have unusual or disrupted topological properties. Rakhimberdina and Murata [40] exploited the simple linear graph [39] model for SZ detection, achieving an accuracy of 80.55% for a binary classification task. The use of the linear model within the graph model has a clear impact on decreasing its computational time. However, the edge construction strategy can be further improved by incorporating techniques to learn the edge weights such as self-attention weight features.

#### 3.1.3. Major Depressive Disorder

Major depressive disorder (MDD) is a mental disease characterised by a depressed mood, diminished interests and impaired cognitive function. Among various neuroimaging techniques, rs-*f*MRI can observe dysfunction in brain connectivity on BOLD signals, and has been used to discriminate between MDD patients and healthy controls. Yao et al. [60] exploited time-varying dynamic information with a temporal adaptive GCN on rs-*f*MRI data to learn the periodic brain status changes to detect MDD. The model learns a data-based graph topology and captures dynamic variations of the brain *f*MRI data, and outperforms traditional GCN [28] and GAT [35] models.

#### 3.1.4. Bipolar Disorder

Bipolar disorder (BD), or manic depression, is a mental health condition that causes extreme mood swings. Functional and structural brain studies have identified quantitative differences between BD and healthy controls; thus, combining modalities may uncover hidden relationships. Yang et al. [44] proposed a graph-attention based method that integrates structural MRI and *f*MRI to detect bipolar disorder. The main challenges in multimodal data fusion are the dissimilarity of the data types being fused and the interpretation of the results. One of the advantages of attention mechanisms is that they allow for the use of variable-sized inputs when focusing on the most important parts of the data to make decisions, which can then be used to interpret the salient input features. The model showed superiority over other machine learning classifiers and alternative GCN formulations.

#### 3.1.5. Brain Responses to Stimulus

Identifying the relationship between brain regions in relation to specific cognitive stimuli has been an important area of neuroimaging research. An emerging approach is to study this brain dynamic using *f*MRI data. To identify these brain states, traditional methods rely on acquisition of brain activity over time to accurately decode a brain state.

Zhang et al. [5] proposed a GCN for classifying human brain activity on 21 cognitive tasks by associating a given window of *f*MRI data with the task used. The GCN takes a short series of *f*MRIs as input (10 s), propagates information among inter-connected brain regions, generates a high-level domain-specific graph representation, and predicts the cognitive state. This model outperforms a multi-class support vector machine classifier in identifying a variety of cognitive states in the HCP dataset [76]. However, the model only incorporates spatial graph convolutions, thus potentially losing the fine temporal information present in the BOLD signal [5].

Identifying the particular brain regions that relate to a specific neurological disorder or cognitive stimuli is also critical for neuroimaging research. GNNs have been widely applied as a graph analysis method. Nodes in the same brain graph have distinct locations and unique identities. Thus, applying the same kernel over all nodes is problematic. Li et al. [61] adopted weighted graphs from *f*MRI and ROI-aware graph convolutional layers to infer which ROIs are important for prediction of cognitive tasks. The model maps regional and cross-regional functional activation patterns for classification of cognitive task decoding in the HCP 900 dataset [76]. The framework is also capable of learning the node grouping and extracts graph features jointly, providing the flexibility to choose between individual-level and group-level explanations.

Deep learning has also been considered a competitive approach for analysing high-dimensional spatio-temporal data such as MEG signals. These signals are captured with 306 sensors (electrodes) distributed across the scalp that record the cortical activation. For reliable analysis, it is critical to learn discriminative low-dimensional intrinsic features. Guo et al. [77] proposed a spectral GCN model that integrates brain connectivity information to predict visual tasks using MEG data. The authors introduced an autoencoder-based network that integrates graph information to extract meaningful representations in an unsupervised manner, and classify whether a subject visualises a face or an object. This work focused on learning a low-dimensional representation from the input of MEG signals (i.e., a dimensionality reduction technique).

### 3.2. Electrical-Based Analysis

This section mainly covers application of graph learning representation on electrical activity including electroencephalogram (EEG), intracranial EEG, electrocardiogram (ECG), and polysomnography (PSG) as summarized in Table 2.

**Table 2:** Summary of GCN approaches adopted to electrical-based analysis and their applications.

Authors	Year	Modality	Application	Dataset
Jang et al. [81]	2019	EEG	Classification: Affective mental states	DEAP [82] (40 classes)
Jang et al. [83]	2018	EEG	Classification: Affective mental states	DEAP [82] (40 classes)
Mathur et al. [84]	2020	EEG	Classification: Seizure detection	University of Bonn [85] (2 classes)
Wang et al. [59] 🟉	2020	EEG	Classification: Seizure detection	University of Bonn [85] (2 classes), SSW-EEG (private) (2 classes)
Covert et al. [86] 🟉	2019	EEG	Classification: Seizure detection	Cleveland Clinic Foundation (private) (2 classes)
Lian et al. [42] †	2020	iEEG	Regression: Seizure prediction (preictal)	Freiburg iEEE (EPILEPSIAE) [87]
Wagh et al. [88]	2020	EEG	Classification: Abnormal EEG	TUH EEG corpus [89], MPI LEMON [90] (2 classes)
Wang et al. [43] †	2020	ECG	Classification: Heart abnormality	HFECGIC [91] (34 classes)
Sun et al. [92]	2020	EGM	Classification: Heart abnormality	EGM open-heart surgery [93] (2 classes)
Jia et al. [45] 🟉†	2020	PSG	Classification: Sleep staging	MASS-SS3 [94] (5 classes)

🟉 GCN with temporal structures for medical diagnostic analysis. † GCN with attention structures for medical diagnostic analysis.

#### 3.2.1. Affective Mental States

**Figure 6:**
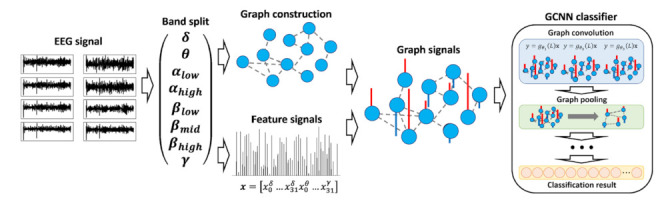
Features are extracted from EEG signals to construct a graph-based architecture and classify mental states. Image adapted from [83].

Brain signals provide comprehensive information regarding the mental state of a human subject. Jang et al. [83] proposed the first method to apply deep learning on graph signals to EEG-based visual stimulus identification. The model converts the EEG into graph signals with appropriate graph structures and signal features as input to GCNs to identify the visual stimulus watched by a human subject. Compared to *f*MRI signals, EEG analysis is limited to observing a smaller number of brain regions (i.e., electrodes) which may not allow for a sufficiently rich graph representation. Thus, the authors create a graph containing both intra-band and inter-band connectivity. This proposed approach is illustrated in Figure 6. Defining the graph connectivity structure for a given task is an ongoing problem and current models still have the limitation that appropriate graph structures need to be manually designed. To address this, Jang et al. [81] proposed an EEG classification model that can determine an appropriate multi-layer graph structure and signal features from a collection of raw EEG signals and classify them. In contrast to approaches that use a pre-defined connectivity structure, this method for learning the graph structure enhances classification accuracy.

#### 3.2.2. Epilepsy

Epilepsy is one of the most prevalent neurological disorders characterised by the disturbance of the brain electrical activity, and recurrent and unpredictable seizures. Machine learning applications have been used for seizure prediction, seizure detection and seizure classification through the analysis of EEG/iEEG signals. CNNs and RNNs have shown success in analysing these signals for Epilepsy related tasks, but they suffer from a loss of neighbourhood information. On the other hand, GCNs represent the relationships between electrodes using edges, and can thus preserve rich connection information.

*Seizure detection* from time-series refers to recognising the ictal activity or that a seizure is occurring (i.e., determine the presence or absence of ongoing seizures). Mathur et al. [84] presented a method for detecting ictal activity using a visibility graph on the EEG by employing a Gaussian kernel function to assign edge weight. A graph discrete Fourier transform is also applied to obtain features which are used in the classification phase. Some works have proven the relationship between epilepsy and EEG components on certain frequencies, and this frequency–domain representation can generate highly interpretable results. Wang et al. [59] introduced a sequential GCN that preserves the sequential information in 1D signals. The model is based on a complex network that represents a 1D signal as a graph [95], in which each data point corresponds to a node and each edge is computed by a connection rule. The authors first transform the time-domain signal using a fast Fourier transform to produce a sequence of frequency–domain features that are aligned in the time domain, from which they develop a graph representation. Then, a GCN is adopted to learn features from the input network to improve the classification performance. By combining the frequency–domain network representation with the GCN, the model can detect conventional seizures in the Bonn dataset [85], and a seizure type known as absence epilepsy from a private dataset. However, multi-channel EEG signals were not considered in the experimental setup. Covert et al. [86] proposed a temporal graph convolutional network (TGCN) which consists of feature extractors that are localized and shared over both time and space. TGCN is inherently invariant to when and where the patterns occur. The authors investigate the benefits of TGCN’s interpretability in terms of assisting clinicians in determining when seizures occur and which areas of the brain are most involved. However, the model is limited to allow for varying graph structures.

*Seizure prediction* aims to predict upcoming seizures or the pre-ictal brain state (i.e., before a seizure). The underlying relationship in the pre-ictal period can be diverse across patients, making it difficult to build a predefined graph that is effective for a large number of patients. To address this, instead of directly using a prior graph, Lian et al. [42] proposed to build a graph based on the influences of relationships. The authors introduced global-local GCNs that jointly learn the structure and connection weights to optimize the task-related learning of iEEG signals. The connections in nodes are updated with attention and gating mechanisms, but the model requires a large volume of data for training.

#### 3.2.3. Abnormal EEG in Neurological Disorders

The application of machine learning techniques to automatically detecting anomalies in medical data are particularly attractive considering the difficulties in consistency and objectivity identifying anomalies. There exist numerous medical anomaly detection tasks, including identifying abnormal EEG recordings of patients with neurological disorders. An assessment is made when analysing an EEG recording to see whether the recorded signal appears to indicate abnormal or regular brain activity patterns.

Recent GCNs have addressed the challenges of learning the spatio-temporal relationships in EEG data. Wagh et al. [88] introduced a GCN that captures both spatial and functional connectivity for multi-channel EEG data to distinguish between “normal” EEGs on patients with neurological diseases and the EEGs of healthy individuals. First, a graph-based representation with its corresponding node-level embedding is extracted from 10-second windows of EEG signals fed through a GCN model. Then, a graph-level embedding is computed using an averaging operation, the output of which is input into a fully connected network to obtain the output class. Finally, a maximum likelihood estimation based on the window-level prediction is adopted to determine if the entire EEG recording was recorded from a particular patient (i.e., subject prediction). Results on two large-scale scalp EEG databases, TUH EEG corpus [89] and MPI LEMON [90], significantly outperform traditional machine learning models. The authors also evaluated the effect of depth on GCNs, and find higher depth offers only a marginal improvement in performance. However, the data from patients and control participates were collected using different systems which may help to distinguish both classes and a feature engineering phase was considered which limits the model’s ability to directly discover the optimal features from the data.

#### 3.2.4. Heart Abnormalities

Electrocardiograms (ECG) are widely used to identify cardiac abnormalities and a variety of methods have been proposed for the classification of ECG signals. However, an ECG record may contain multiple concurrent abnormalities and current deep learning methods may ignore the correlations between classes, and looks at each class independently. This can be addressed via graph-based representations.

The GAT architecture has matched or surpassed state-of-the-art results across graph learning benchmarks. Still, it is designed to only classify nodes within a single network, and it can only deal with binary graphs. Wang et al. [43] proposed a multi-label weighted graph attention network to classify 34 kinds of electrocardiogram abnormalities. In this model, ECG features are extracted from a CNN (1D ResNet). The features of each class are fed into an improved GAT by integrating a co-occurrence weight with masked attentional weights. The weighted GAT helps capture the relationships within the ECG abnormalities. Then, the features learnt by the CNN and GAT are concatenated to output the probability of each class.

The epicardial electrogram (EGM) is measured on the heart’s surface and has been used to analyse atrial fibrillation, a clinical arrhythmia correlated with stroke and sudden death. Conventional signal processing methods are less suitable for joint space time and frequency domain analysis. Sun et al. [92] represented the spatial relationships of epicardial electrograms through a graph to formulate a high-level model for atrial activity. The authors evaluated the spatio-temporal variation of EGM data with a graph-time spectral analysis framework and identified spectral differences between normal heart rhythms and atrial fibrillation from EGM signals taken during open heart surgery [93].

#### 3.2.5. Sleep Staging

Sleep stage classification, the process of segmenting a sleep period into epochs, is essential for clinical assessment of sleep disorders including insomnia, circadian rhythm disorders, and sleep-related breathing and movement disorders [96], which may lead to serious health problems affecting quality of life. Sleep staging analysis is conducted through the analysis of electro-graphic measurements of the brain, eye movement, chin muscles, cardiac and respiratory activity, and is collected with a polysomnography (PSG). The manual determination of sleep stages on PSG records is a complex, costly, and problematic process that requires expertise. Although traditional CNN and RNN models can achieve high accuracy for automatic sleep stage classification, the models ignore the connections among brain regions and capturing the transition between sleep stages continues to be challenging. Sleep experts identify one sleep stage according to both EEG patterns and the class label of its neighbours. To address these challenges, Jia et al. [45] adopted an adaptive graph connection representation with attention, ST-GCN [46], for automatic sleep stage classification and to capture sleep transition rules temporally. First, the pairwise relationship between nodes (EEG channels) is constructed dynamically; then, a ST-GCN model with attention is adopted to extract both spatial and temporal features. Experimental results in classifying five sleep stages on the PSG dataset MASS-SS3 [94] achieves the best performance compared to SVM, CNN and RNN baselines.

### 3.3. Anatomical Structure Analysis (Classification and Prediction)

This section covers application of graph learning representation on anatomical structure analysis for classification with input data such as magnetic resonance image (MRI), T1 weighted image (T1W1), difussion MRI (DMRI), computed tomography (CT), X-ray and ultrasound (US) as summarized in Table 3.

**Table 3:** Summary of GCN approaches adopted for anatomical structure analysis and their applications (Group 1).

Authors	Year	Modality	Application	Dataset
Ma et al. [97] †	2020	MRI	Classification: Alzheimer’s disease	ADNI [98] (2 classes)
Huang et al. [99]	2020	MRI/*f*MRI	Classification: Alzheimer’s disease	ADNI [100] (3 classes)
Huang et al. [18]	2020	MRI	Classification: Alzheimer’s disease	ADNI [100] (3 classes), TADPOLE [101] (3 classes)
Yu et al. [102]	2020	MRI	Classification: Alzheimer’s disease/MCI	ADNI [100] (3 classes)
Gopinath et al. [20]	2020	MRI	Classification: Alzheimer’s disease	ADNI [100] (2 classes)
Zhao et al. [103]	2019	MRI	Classification: Alzheimer’s disease/MCI	ADNI [100] (2 classes)
Wee et al. [104]	2019	MRI	Classification: Alzheimer’s disease	ADNI [100] (2 classes), Asian cohort (private) (2 classes)
Kazi et al. [67]	2019	MRI	Classification: Alzheimer’s disease	TADPOLE [101] (3 classes)
Song et al. [105]	2019	MRI	Classification: Alzheimer’s disease	ADNI [100] (4 classes)
Gopinath et al. [36]	2019	MRI	Classification: Alzheimer’s disease	ADNI [100] (2 classes)
Guo et al. [106]	2019	PET	Classification: Alzheimer’s disease	ADNI${}_{2}$ [107] (2/3 classes)
Parisot et al. [15]	2018	MRI	Classification: Alzheimer’s disease	ADNI [100] (3 classes)
Parisot et al. [72]	2017	MRI	Classification: Alzheimer’s disease	ADNI [100] (3 classes)
Xing et al. [53] 🟉	2019	T1WI/*f*MRI	Classification: Alzheimer’s disease/EMCI	ADNI [98] (2 classes)
Zhang et al. [108]	2018	sMRI/DTI	Classification: Parkinson’s disease	PPMI [109] (2 classes)
McDaniel and Quinn [110] †	2019	sMRI/dMRI	Classification: Parkinson’s disease	PPMI [109] (2 classes)
Zhang et al. [47] †	2020	sMRI/dMRI	Classification: Parkinson’s disease	PPMI [109] (2 classes)
Yang et al. [37]	2019	MRI	Classification: Brain abnormality	Brain MRI images (private) (2 classes)
Wang et al. [111]	2020	CT	Classification: COVID-19 detection	Chest CT scans (private) (2 classes)
Yu et al. [112]	2020	CT	Classification: COVID-19 detection	Hospital of Huai’an City (private) (2 classes)
Wang et al. [113]	2021	CT	Classification: Tuberculosis	Chest CT scans (private) (2 classes)
Hou et al. [114] †	2021	X-ray	Classification: Chest phatologies	IU X-ray [115] (14 classes), MIMIC-CXR [116] (14 classes)
Zhang et al. [117] †	2020	X-ray	Classification: Chest phatologies	IU-RR [115] (20 classes)
Chen et al. [118]	2020	X-ray	Classification: Chest phatologies	ChestX-ray14 [119] (14 classes), CheXpert [120] (14 classes)
Zhang et al. [121]	2021	X-ray	Classification: Breast Cancer	mini-MIAS (mammogram) [122] (6 classes)
Du et al. [123]	2019	X-ray	Classification: Breast cancer	INbreast (full field digital mammogram) [124] (2 classes)
Yin et al. [125]	2019	US	Classification: Kidney disease	Children’s Hospital of Philadelphia (private) (2 classes)
Liu et al. [126]	2020	MRI	Regression: Relative brain age	Preterm MRI (private)
Gopinath et al. [20]	2020	MRI	Regression: Relative brain age	ADNI [100]
Gopinath et al. [36]	2019	MRI	Regression: Relative brain age	ADNI [100]
Chen et al. [127]	2020	DMRI	Regression: Brain data	BCP [128]
Kim et al. [129]	2019	DMRI	Regression: Brain data	DMRI neonate (private)
Hong et al. [130]	2019	DMRI	Regression: Brain data	DMRI infant (private)
Hong et al. [7]	2019	DMRI	Regression: Brain data	HCP [131]
Hong et al. [132]	2019	DMRI	Regression: Brain data	HCP [131]
Cheng et al. [133]	2020	MRF	Regression: Brain data	3D MRF (private)

🟉 GCN with temporal structures for medical diagnostic analysis. † GCN with attention structures for medical diagnostic analysis.

#### 3.3.1. Alzheimer’s Disease

Alzheimer’s disease (AD) is an irreversible brain disorder which destroys memory and cognitive ability. There is as yet no cure for AD and monitoring its progress [Cognitively Normal (CN), Significant Memory Concern (SMC), Mild Cognitive Impairment (MCI) (including early MCI (EMCI), and late MCI (LMCI)) and AD] is essential to adjust the therapy plan for each stage.

Similar to Autism Spectum Disorder (ASD), GCNs can be used to classify subjects into healthy or AD. Parisot et al. [15] constructed a population graph by integrating subject-specific imaging (MRI) and pairwise interactions using non imaging (phenotypic) data, then fed the sparse graph to a GCN to perform a semi-supervised node classification. Their experiments on the ADNI dataset for AD classification (conversion from (MCI) to AD) showed a high performance in comparison to a non-graph method [134]. In addition, comparing to their prior work [72], they showed a better graph structure (combining APOE4 gene data and eliminating AGE information) that could increase the accuracy of binary classification of AD on the ADNI dataset.

Huang et al. [18] applied their edge-variational GCN (EV-GCN) method to the ADNI dataset for AD classification (the data were prepared in the same manner as Parisot [72]). In addition, they applied their method on TADPOLE [101] which is a subset of ADNI for classifying subjects into cognitive normal, MCI, and AD. For TADPOLE, the authors constructed a graph by using the segmentation features inferred from MRI and PET data, phenotypic data, APOE, and FDG-PET biomarkers. Their results on both datasets showed a high performance in comparison to Parisot [72] and Inception GCN [67].

Zhao et al. [103] developed a GCN based method to predict MCI (EMCI vs. NC, LMCI vs. NC and LMCI vs. EMCI) from rs-*f*MRI. They constructed the MCI-graph using both imaging data extracted from rs*f* MRI and non-imaging data including gender and collection device information. They classified the nodes in the generated MCI-graph using GCN and Cheby-GCN and compared the results with a Ridge, a random forest classifier and a multilayer perceptron, and demonstrated a high performance for Cheby-GCN over those methods.

Xing et al. [53] proposed a model consisting of dynamic spectral graph convolution networks (DS-GCNs) to predict early mild cognitive impairment (EMCI), and two assistive networks for gender and age to provide guidance for the final EMCI prediction. They constructed graphs using T1-weighted and *f*MRI images from the ADNI [98] dataset. Apart from predicting age and gender for EMCI prediction, their model used an LSTM which could extract temporal information related to the EMCI prediction.

Yu et al. [102] used a multi-scale enhanced GCN (MSE-GCN) and applied it to a population graph which was built by combining imaging data(rs-*f*MRI and diffusion tensor imaging (DTI)) and demographic relationships (e.g., gender and age) to predict EMCI. This resulted in better performance in comparison to the prior methods of Zhao et al. [103] and Xing et al. [53]. Huang et al. [99] processed multi-modal data, MRI and rs-*f*MRI, to identify EMCI. First, feature representation and multi-task feature selection are applied to each input. Then, a graph was developed using imaging and non-imaging (phenotypic measures of each subject) data. Finally, a GCN was used to perform the EMCI identification task from the ADNI dataset [100].

Song et al. [105] built a structural connectivity graph from DTI data from the ADNI imaging dataset and implemented a multi-class GCN classifier for the four class classification of subjects on the AD spectrum. The receiver operating characteristic (ROC) curve was compared between GCN and SVM classifiers for each class and demonstrated the capability of GCN over SVM (which relies on a predefined set of input features) for AD classification.

For the subject-specific aggregation of cortical features (MRI images), Gopinath et al. [20,36] proposed an end-to-end learnable pooling strategy. This method is a two-stream network, one for calculating latent features for each node of the graph, and another for predicting node clusters for each input graph. The learnable pooling approach can handle graphs with a varying number of nodes and connectivity. The results of their binary classification on the ADNI dataset [98] for NC vs. AD, MCI vs. AD, and NC vs. MC, showed the value of leveraging geometrical information in the GCN.

Guo et al. [106] constructed a graph from the ROI of each subject’s PET images from the ADNI${}_{2}$ dataset [107], and proposed a PETNet model based on GCNs for EMCI, LMCI, or NC prediction. The proposed method is computationally inexpensive and more flexible in comparison to voxel-level modeling.

Ma et al. [97] proposed an Attention-Guided Deep Graph Neural (AGDGN) network model to derive both structural and temporal graph features from the ADNI dataset [98]. This dataset contains four classes; however, due to a shortage of data to train this model, they combined CN and SMC to form the CN group, and MCI and AD to form the AD group. This resulted in a two-class classification problem. They used an attention-guided random walk (AGRW) process to extract noise-robust graph embeddings. Their results indicated that the identified AD characteristics detected by the proposed model aligned with those reported by clinical studies.

To reduce the burden of creating a reliable population-specific classifier from scratch, generalization of classifiers to other datasets or populations, especially those with a limited sample size, is critical. Wee et al. [104] employed a spectral graph CNN that incorporates the cortical thickness and geometry from MRI scans to identify AD. To demonstrate the generalisation and the feasibility to transfer classifiers learned from one population to another, the authors trained on a sizable caucasian dataset from the ADNI cohort [100], and evaluate how well the classifier can predict the diagnosis of an Asian population. To transfer the spectral graph-CNN model, the model that worked best on the ADNI cohort’s testing set was fine-tuned on the training set of the Asian population. The performance of the fine-tuned model was then assessed using the testing set of the Asian cohort.

#### 3.3.2. Parkinson’s Disease

Parkinson’s Disease (PD) is a neurological disorder characterized by motor and non-motor impairments. Motor deficits include bradykinesia, rigidity, postural instability, tremor, and dysarthria; and non-motor deficits include depression, anxiety, sleep disorders, and slowing of thought. Neuroimaging research using structural, functional, and molecular modalities have also shed light on the underlying mechanism of Parkinson’s disease. Many imaging based biomarkers have been demonstrated to be closely related to the progression of PD. Zhang et al. [108] developed a framework for analyzing neuroimages using GCNs to learn similarity metrics between subjects with PD and HC using data from the PPMI dataset [109]. Structural brain MRIs are divided into a set of ROIs where each region is treated as a node on an undirected and weighted brain geometry graph. The authors showed the effectiveness of GCNs to learn features from similar regions and proposed a multi-view structure to fuse different MRI acquisitions. However, in this approach, temporal dependency is not considered.

McDaniel and Quinn [110] addressed the issue of analyzing multi-modal MRI data together by implementing a GAT layer to perform whole-graph classification. Instead of making predictions based on pairwise examples, GCNs predict the class of neuroimage data directly.

The features on each vertex must be pooled to generate a single feature vector for each input in order to convert the task from classifying each node to classifying the entire graph. The self-attention mechanism in GAT is used to compute the importance of graph vertices in a neighbourhood, allowing for a weighted sum of the vertices’ features during pooling. The results of combining diffusion and anatomical data from the PPMI dataset [109] with the proposed model outperforms baseline algorithms on the features constructed from the diffusion data alone. The GAT attention layer also enables the possibility to interpret the magnitude of each node’s attention weight as the relative importance of a brain area for discriminating PD participants.

Current brain network methods either ignore the intrinsic graph topology or are designed for a single modality. To address these challenges, Zhang et al. [47] proposed a graph representation to fuse functional (*f*MRI) and structural brain networks (MRI). The cross-modality relationships and encoding is generated by an encoder–decoder process. The authors adopted the idea of the GAT model for a dynamic adjustment of the weights. Here, three aggregation mechanisms are dynamically combined (graph attention weight, the original edge weight, and the binary weight) through a multi-stage graph convolutional kernel. This model achieves the best prediction performance compared to CNN-based and graph-based approaches. The model is capable of localizing 10 key regions associated with PD classification via a saliency map (e.g., the bilateral hippocampus and basal ganglia which are structures conventionally conceived as PD biomarkers).

#### 3.3.3. Brain Abnormality

The ability to correctly recognize anomalous data is a deciding and crucial factor, so a highly accurate abnormality detection model is needed. Yang et al. [37] proposed a synergic graph-based model for a normal/abnormal classification of brain MRI images. The synergic deep learning method [38] can address the challenges faced by a GCN in distinguishing intra-class variation and inter-class similarity. To improve the efficiency, the authors first extract the ROI of the image and use segmentation models as input to the model. The network consists of a dual GCN component (a pair of GCN models of identical construction) and a synergic training component. The synergic training component is used to predict whether a pair of images in the input layer belong to the same class and gives feedback if there is a synergic error.

#### 3.3.4. Coronavirus 2 (SARS-CoV-2 or COVID-19)

Early diagnosis of coronavirus is significant for both infected patients and doctors providing treatments. Viral nucleic acid tests and CT screening are the most widely used techniques to detect pneumonia which is caused by the virus, and thus to make a diagnosis. Although CNNs have demonstrated a powerful capability to extract and combine spatial features from CT images, they are hindered because the underlying relationships between each element are ignored. Thus, GCNs are receiving attention in the analysis of COVID-19 patient CT images. Yu et al. [112] develop a graph framework that combined a graph representation with a CNN suitable for COVID-19 detection. A CNN model is used for feature extraction and graphs of the extracted features are constructed. Each feature is taken as one node of the graph while the edges between nodes are built according to the top *k* neighbours with the highest similarity. The distance between nodes is measured by the Euclidean distance, while edges are quantified by the adjacency matrix. Classification performance into healthy and infected classes shows promising results, but the search domain of the size of batch and the number of neighbours needs further exploration.

Wang et al. [111] also proposed an improved CNN that is combined with a GCN for higher classification accuracy. CNNs yield an individual image-level representation and the GCN focuses on a relation-aware representation. These representations are fused at the feature-level for COVID-19 detection from CT images. Although the model outperforms traditional CNN architectures, the method is limited in handling other modalities such as chest X-rays which are widely used to assist COVID-19 detection due to its availability, quick response, and cost-effective nature.

#### 3.3.5. Tuberculosis

Tuberculosis (TB) is an infectious decease that can affect different organs such as abdomen and nervous system, but normally infects the lungs and is known as pulmonary TB (PTB). Two main categories of PTB are primary pulmonary tuberculosis (PPT) and secondary pulmonary tuberculosis (SPT). Wang et al. [113] investigated the GCN model to recognize the SPT as many PTB cases are turned to be an SPT type. They proposed a rank-based pooling neural network (RAPNN) by which individual image-level features can be extracted, then integrated the GCN to RAPNN and built a new model called GRAPNN to identify the SPT. The explainability of the proposed model was analyzed using Grad-ACM, and their results outperformed SOTA including CNN models.

#### 3.3.6. Chest Pathologies

Chest X-ray imaging has been used to assist clinical diagnosis and treatment of several thoracic diseases where an individual image might be associated with multiple abnormalities, necessitating a multi-label image classification task. Several approaches have transformed a multi-label classification problem into multiple disjoint binary classification problems without acknowledging any label correlations. Abnormalities may be closely linked and label co-occurrence and interdependencies between these abnormal patterns (i.e., strong correlations among pathologies) are important for diagnosis.

**Figure 7:**
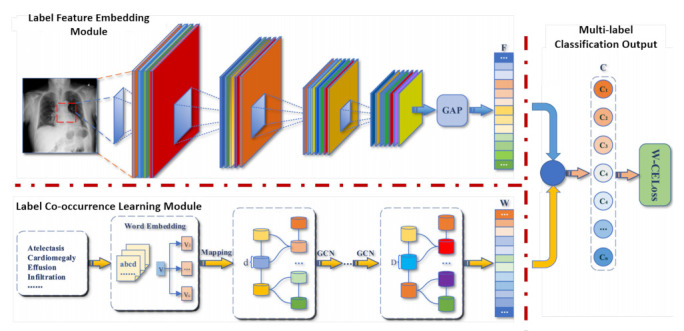
A GCN-based label co-occurrence learning framework to explore potential abnormalities with the guidance of semantic information, including the pathology co-occurrence and interdependency. Image adapted from [118].

To address the limitations of current models that lack a robust ability to model label co-occurrences and capture interdependencies between labels and regions, Chen et al. [118] introduced a label co-occurrence learning framework based on GCNs to find dependencies between pathologies from chest X-ray imaging. This framework consists of two modules, an image feature embedding module that learns high-level features from images and a label co-occurrence learning module that classifies different pathology categories. In the framework which is illustrated in Figure 7, each pathology is illustrated with semantic vectors via a word embedding, and the graph representation is learned from the co-occurrence matrix of training data. The classifiers are combined with image-level features to adaptively revise prediction beliefs for each pathology in two large-scale chest X-ray datasets, ChestX-ray14 [119] and CheXpert [120]. Although this approach models the correlations among disease labels, the utilization of medical reports paired with radiology images was not covered.

Zhang et al. [117] adapted attention mechanisms and GCNs to learn graph embedded features to improve classification and report generation. In this approach, a CNN feature extractor and attention mechanism are used to compute initial node features. Then, a graph is developed with prior knowledge on chest findings to learn discriminatory features and the relationship between them for classifying disease findings. Each node in the graph corresponds to a finding category. Once the classification network is trained, a two-level decoder with recurrent units (LSTMs) is trained to generate reports. The decoder learns to attend to different findings on the graph, and focuses on one concept in each sentence. The performance demonstrated with the IU-RR dataset [115] indicates that graphs with prior knowledge help to generate more accurate reports. Hou et al. [114] employed a transformer encoder as the feature-fusion model of both visual features and label embeddings (semantic features pre-trained on large free-text medical reports). These features are fed to a GCN model which is built as the knowledge graph to model the correlations among different thoracic diseases. The graph is constructed by a data-driven method from medical reports, with primary and auxiliary nodes that correspond to disease labels and other medical labels, respectively. However, its extension to handle other domains is limited because the graph is not built automatically.

#### 3.3.7. Breast Cancer

**Figure 8:**
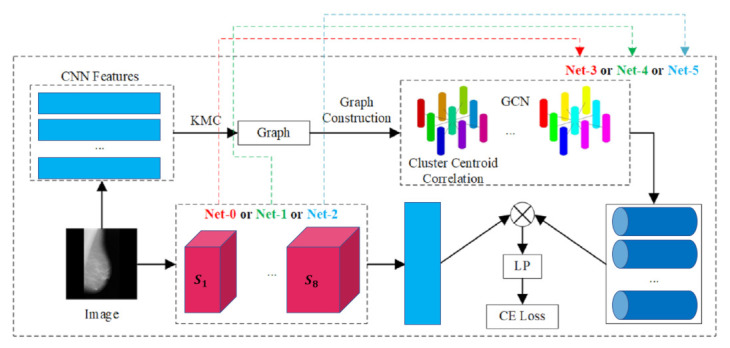
Illustration of the framework that combines CNN and GCN features. Bottom row shows the CNN pipeline to extract image-based features while the top row illustrates the GCN pipeline to learn the interactions. Image adapted from [121].

For abnormal breast tissue detection, the aim is to not only learn the image-level representation automatically, but also the relation-aware representation to more accurately detect abnormal masses using mammography. Zhang et al. [121] fused a CNN pipeline with a GCN pipeline to attain superior performance in classifying six abnormal types in the mini-MIAS dataset [122]. First, a CNN extracts individual image-level features; then, a GCN estimates a relation-aware representation. These features are combined via a dot product and a linear projection with trainable weights. This framework is illustrated in Figure 8. Although the proposed model achieves high accuracy when analysing mammographic data, further optimization on larger datasets was not considered and other combination mechanisms of GCN and CNN should be assessed.

In clinical practice, experts review medical images by zooming into ROIs for a close-up examination. Thus, Du et al. [123] model the zoom-in mechanism of radiologists’ operation with a hierarchical graph-based model to detect abnormal lesions with full field digital mammogram (FFDM) images from the INbreast dataset [124]. A pre-trained CNN trained on lesion patches is used to extract features and a GAT model classifies nodes to predict whether to zoom or not into the next level to predict a benign or malignant mammogram. By adding the zoom-in mechanism, model interpretability is improved. However, the INbreast dataset is relatively small making this method difficult to assess, and a new loss is required to supervise the zoom-in mechanism.

#### 3.3.8. Kidney Disease

In nephrology, ultrasound (US) data are widely used for diagnostic studies of the kidneys and urinary tract and the anatomic measurement of the renal parenhymal area is correlated with kidney function. Machine learning studies have shown promising performance for the tasks of segmentation and classification of US data; however, kidney disease diagnosis is still a challenging task due to the heterogeneous appearance of multiple 2D US scans of the same kidney from different views. Multiple instance learning has been used to estimate instance-level classification probabilities and fuse them to generate a bag-level classification probability, but correlation between instances has not been well explored. To improve these methods, Yin et al. [125] introduced a graph-based methodology to detect children with congenital anomalies of the kidneys and urinary tract in 2D US images. A CNN is used to learn informative US image features at the instance level and a GCN is used as a permutation-invariant operator to further optimize the instance-level CNN features by exploring potential correlations among different instances of the same bag. The authors also adopted attention-based multiple instance learning pooling to learn a bag-level classifier using an instance-level supervision to enhance the learning of instance features and the bag-level classification.

#### 3.3.9. Relative Brain Age

Predicted brain age is a meaningful index that characterises the current status of brain development which may be associated with functional brain abilities in the future. Measurements of morphological changes, including sulcal depth and cortical thickness, can be key features for brain age prediction. Traditional approaches applied to surface morphological features have not taken into account the topology of surfaces, which is defined with meshes. Therefore, CNN-based methods may not be appropriate for the analysis of cortical surface data. A relative brain age has been used as a metric computed as the predicted age minus the true age of the subject. Liu et al. [126] exploited the brain mesh topology as a sparse graph to predict brain age from MRIs for preterm neonates using vertex-wise cortical thicknesses and sulcal depth as input to a GCN. This model enables the convolutional filtering of input features through the surface topology in the context of spectral graph theory. The GCN predicted the ages of preterm neonates better than machine learning and deep learning methods that did not use surface topological knowledge. The authors also generated cortical sub-meshes that represent brain regions to predict which region estimates the age more accurately, and if they are associated with brain functional abilities in the future. As discussed previously in the subsection of AD analysis, Gopinath et al. [20,36] also demonstrated their adaptive graph convolution pooling in a regression problem where the brain age is estimated using the geometry of the brains with point-wise surface-based measurements. The model is trained using data labeled as normal cognition from the ADNI dataset [100], and the graph model uses cortical thickness, sulcal depth and spectral information to predict the brain age.

#### 3.3.10. Brain Data Prediction

Diffusion MRI (DMRI) provides unique insights into the developing brain, owing to its sensitivity examination of brain tissue microstructures and white matter properties which are useful for diagnosis of brain disorders. However, DMRI suffers from long acquisition times and is more susceptible to low signal-to-noise ratio, motion artifacts, and partial volume effects. Missing data are also a common problem in longitudinal studies due to unsuccessful scans and subject dropouts, and the high variability in diffusion wave-vector sampling (q-space) makes the longitudinal prediction of DMRI data a challenging task. Therefore, some methods have been developed for DMRI reconstruction.

**Figure 9:**
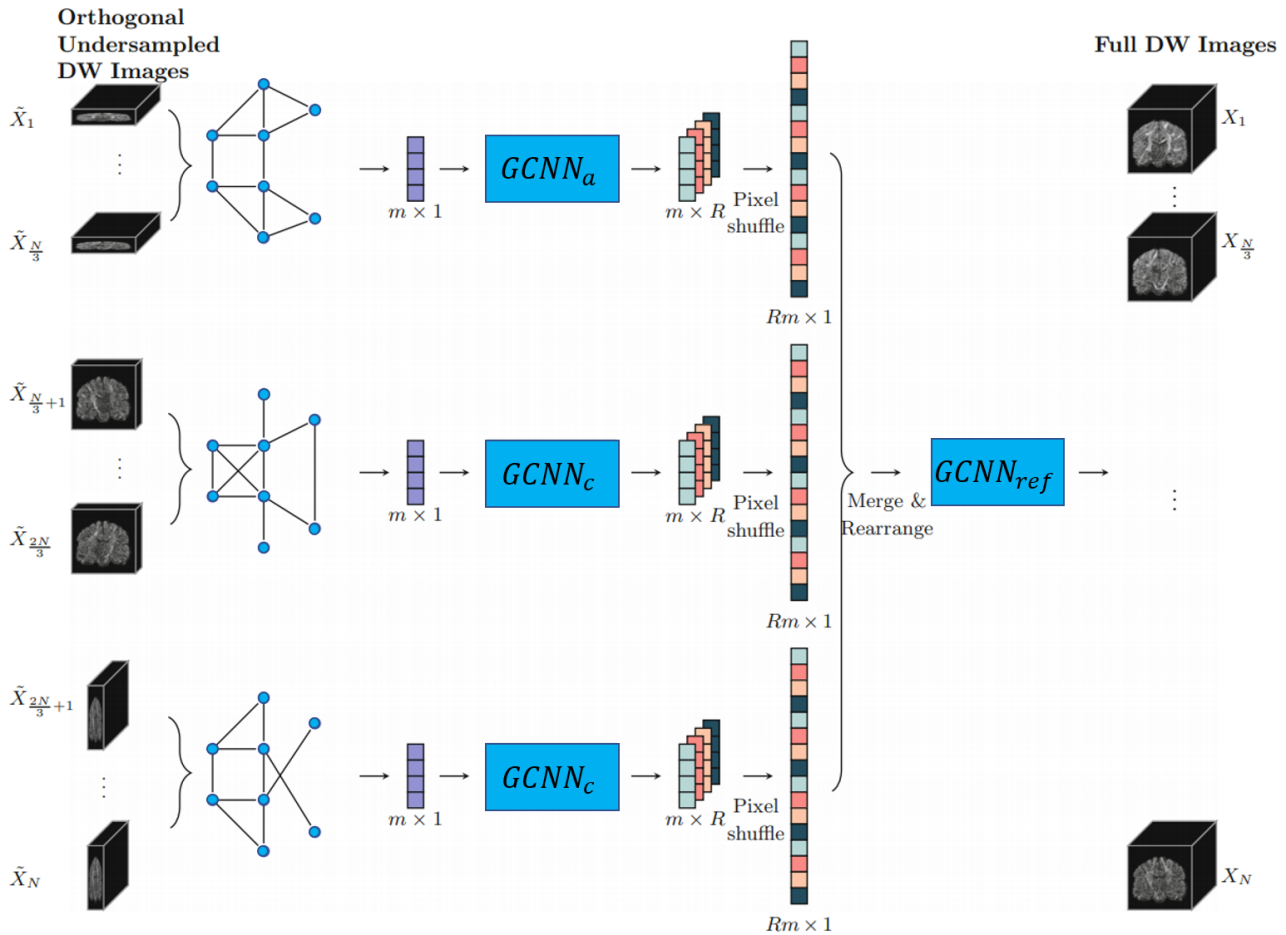
Individual GCNs model the axial, coronal and sagitall scan direction. A refinement GCN is used to generate the proposed super-resolution reconstruction. Image adapted from [132].

To improve acquisition speed, Hong et al. [7] introduced a method for DMRI reconstruction from under-sampled slide data, where only a sub-sample of equally-spaced slices are used to acquire a full diffusion-weighted (DW) image volume. A GCN learns the nonlinear mapping from the sub-sampled to full DW image, and spatio-angular relationships are considered when constructing the graph. To improve perceptual quality, the GCN is employed as the generator in a generative adversarial network. The same authors [132] proposed a super-resolution reconstruction framework based on an orthogonal under-sampling scheme to increase complementary information within the under-sampled DW volume. The set of wave-vectors is divided into three subsets of scan directions (axial, coronal, or sagittal) and they are fitted to individual GCNs. A refinement GCN is used to generate the final DW volume by considering the correlation across scan directions as illustrated in Figure 9. These graph-based methods outperform traditional interpolation methods and 3D UNet based reconstruction methods.

Kim et al. [129] introduced a graph-based model for longitudinal prediction of DMRI data by considering the relationship between sampling points in the spatial and angular domains, i.e., a graph-based representation of the spatio-angular space. Then, the authors implemented a residual learning architecture with graph convolutions to capture brain longitudinal changes to predict missing DMRI data over time in a patch-wise manner. The proposed model showed improved performance in predicting missing DMRI data from neonate images so that longitudinal analysis can be performed. Hong et al. [130] also proposed a GCN-based method for predicting missing infant brain DMRI data. This model exploits information from the spatial domain and diffusion wave-vector domain jointly for effective prediction. Generative adversarial networks (GANs) are also adopted to better model the nonlinear prediction mapping and performance improvement. Here, the generator estimates the source image and the discriminator distinguishes the source image from the estimated one, where the generator is the GCN and the discriminator is developed via consecutive graph convolutional layers. However, the model cannot predict missing DMRI data for arbitrary time points.

Although DMRI is a powerful tool for the characterization of tissue microstructures, several microstructure models need DMRI data densely sampled in *q*-space that is defined by the number of acquired diffusion-weighted images. Traditional deep learning models learn the relationship between sparsely sampled *q*-space data and high-quality microstructure indices estimated from densely sampled *q*-space, but these models do not consider the *q*-space data structure. Chen et al. [127] adopted GCNs to estimate tissue microstructure from DMRI data represented as graphs. The graph encodes the geometric structure of the *q*-space sampling points which harnesses information from angular neighbours to improve estimation accuracy. Results on the baby connectome project dataset [128] demonstrated high-quality intra-cellular volume fraction maps that are close to the gold standard.

Most quantitative MRI methods are comparatively slow and provide a single tissue property at a time, which limit their adoption in routine clinical settings. Magnetic resonance fingerprinting (MRF) is a rapid and efficient quantitative imaging method that has been used for simultaneous quantification of multiple tissue properties in a single acquisition [135]. 2D MRF has been extended to 3D using stack-of-spirals acquisitions, but the high spatial resolution and volumetric coverage prolong the acquisition time. Cheng et al. [133] adopted a GCN to accelerate high-resolution 3D MRF acquisition by interpolating the under-sampled data along the slice-encoding direction. A network is further applied to generate tissue property maps. For efficient tissue quantification, a UNet is used along the temporal domain.

### 3.4. Anatomical Structure Analysis (Segmentation)

Among different medical image segmentation and labeling methods, graph-based methods are showing promising results in clinical applications. Graph-based segmentation approaches play an important role in medical image segmentation. A graph maps pixels or regions in the original image to nodes in the graph. Then, the segmentation problem can be transformed into a labeling problem which requires assigning the correct label to each node according to its properties [136]. GCNs can propagate and exchange the local short-range information through the whole image to learn the semantic relationships between objects. We cover only application with evidence of graph representation learning in anatomical structures including the vasculature system and organs as summarized in Table 4.

**Table 4:** Summary of GCN approaches adopted for anatomical structure analysis and their applications (Group 2).

Authors	Year	Modality	Application	Dataset
Wolterink et al. [137]	2019	CTA	Segmentation: Coronary artery	Coronary Artery Stenoses Detection [138]
Zhai et al. [139]	2019	CT	Segmentation: Pulmonary artery-vein	Sun Yat-sen University Hospital (private)
Noh et al. [24]	2020	FA / Fundus	Segmentation: Retinal vessels	Fundus and FA (private), RITE A/V [140]
Shin et al. [141]	2019	RGB/FA/XRA	Segmentation: Retinal vessels	DRIVE [142], STARE [143], CHASE_DB1 [144], HRF [145]
Chen et al. [146]	2020	MRA	Segmentation: Intracranial arteries	MRA [147], UNC [148]
Yao et al. [149]	2020	CTA	Segmentation: Head and neck vessels	Head and neck CTA (private)
Lyu et al. [150]	2021	MRI	Segmentation: Cerebral cortex	NORA-pediatric [151], HCP-adult [152]
Gopinath et al. [23]	2020	MRI	Segmentation: Cerebral cortex	MindBoggle [153]
Gopinath et al. [20]	2020	MRI	Segmentation: Cerebral cortex	MindBoggle [153]
Hao et al. [22]	2020	T1WI	Segmentation: Cerebral cortex	University of California Berkeley Brain Imaging Center (private)
He et al. [154] †	2020	MRI	Segmentation: Cerebral cortex	MindBoggle [153]
Gopinath et al. [155]	2019	MRI	Segmentation: Cerebral cortex	MindBoggle [153]
Wu et al. [21]	2019	MRI	Segmentation: Cerebral cortex	Neonatal brain surfaces (private)
Parvathaneni et al. [156]	2019	T1WI	Segmentation: Cerebral cortex	Cortical surface (private)
Zhao et al. [19]	2019	MRI	Segmentation: Cerebral cortex	Infant brain MRI (private)
Cucurull et al. [157] †	2018	MRI	Segmentation: Cerebral cortex	HPC mesh [76,158]
Selvan et al. [8]	2020	CT	Segmentation: Pulmonary airway	Danish Lung Cancer Screening trial [159]
Juarez et al. [41]	2019	CT	Segmentation: Pulmonary airway	Danish Lung Cancer Screening trial [159]
Selvan et al. [160]	2018	CT	Segmentation: Pulmonary airway	Danish Lung Cancer Screening trial [159]
Yan et al. [161]	2019	MRI	Segmentation: Brain tissue	BrainWeb18 [162], IBSR18 [163]
Meng et al. [164,165] †	2020	FA	Segmentation: Optic disc/cup	Refuge [166], Drishti-GS [167], ORIGA [168], RIGA [169], RIM-ONE [170]
Meng et al. [164,165] †	2020	US	Segmentation: Fetal head	HC18-challenge [171]
Soberanis-Mukul et al. [172,173]	2020	CT	Segmentation: Pancreas and Spleen	NIH pancreas [174], MSD-spleen [175]
Tian et al. [25]	2020	MRI	Segmentation: Prostate cancer	PROMISE12 [176], ISBI2013 [177], in-house (private)
Chao et al. [178]	2020	CT/PET	Segmentation: Lymph node gross tumor	Esophageal radiotherapy (private)

GCN with temporal structures for medical diagnostic analysis. † GCN with attention structures for medical diagnostic analysis.

#### 3.4.1. Vasculature Segmentation

*Coronary arteries:* Quantitative examination of coronary arteries is an important step for the diagnosis of cardiovascular diseases, stenosis grading, blood flow modeling and surgical planning. Coronary CT angiography (CTA) images are used to determine the anatomical or functional severity of coronary artery stenosis (i.e., a narrowing in the artery). Methods for coronary artery segmentation are related to lumen (i.e., vessel wall) segmentation. Deep learning-based segmentation predicts dense segmentation probability maps (voxel-based segmentation methods), or incorporates a shape prior to exploiting the fact that vessel segment has a roughly tubular shape. Thus, the segmentation can be obtained by deforming the wall of this tube to match the visible lumen in the CTA image.

Graph convolutional networks have also been investigated by Wolterink et al. [137] for coronary artery segmentation in CTA. The authors proposed to use GCNs to directly optimize the position of the tubular surface mesh vertices. The locations of these tubular surface mesh vertices were directly optimized using vertices on the coronary lumen surface mesh as graph nodes. Predictions for vertices rely on both local features and representations of adjacent vertices on the surface. The authors demonstrated that, by considering the information from neighbouring vertices, the GCN generates smooth surface meshes without post-processing.

*Pulmonary arteries and veins:* Separation of pulmonary arteries and veins is challenging due to their similarity in morphology and the complexity of their anatomical structures. Using chest CT, vasculopathy or disease affecting blood vessels can be quantified automatically by detecting pulmonary vessels. Zhai et al. [139] proposed a method that links CNNs with GCNs and can be trained in an end-to-end manner. The model includes both local image and graph connectivity features for pulmonary artery-vein separation. Instead of using entire graphs, the authors proposed a batch-based technique for CNN-GCN training and validation. In this approach, the size of the adjacency matrix can be reduced as the nodes in the GCN are from sub-sampled pixel or voxel grids.

*Retinal vessels:* Assessment of retinal vessels is needed to diagnose various retinal diseases including hypertension and cerebral disorders. Fluorescein angiography (FA) and fundus images have been used for artery and vein classification and segmentation techniques because arteries and veins are highlighted separately at different times due to the flow of the fluorescent dye through the vessels. Noh et al. [24] combined both the fundus image sequence and FA image as input for artery and vein classification. The proposed method comprises a feature extractor CNN for the input images and a hierarchical connectivity GNN based on Graph UNets [179] to incorporate higher order connectivity into classification. Shin et al. [141] also incorporated a GCN into a unified CNN architecture for 2D vessel segmentation on retinal image datasets. A CNN was trained for feature extraction of local appearance and vessel probabilities and a GCN was trained to predict the presence of a vessel based on global connectivity of vessel structures. The vessel segmentation is generated by using the relationship between the neighbourhood of vessels pixels. This is based on the local appearance of vessels instead of vessel structure. The method achieved competitive results, but the classifier cannot be trained end-to-end.

*Intracranial arteries:* Characterization of intracranial arteries (ICA), including labeling each artery segment with its anatomical name, is beneficial for clinical evaluation and research. Many natural and disease related (e.g., stenosis) variations in ICA are challenging for automated labeling. Chen et al. [146] proposed a GNN model with hierarchical refinement to label arteries in magnetic resonance angiography (MRA) data by classifying types of nodes and edges in an attributed relational graph. GNNs based on the message passing framework [180] take a graph with edge and node features as input and return a graph with other features for node and edge types.

*Head and neck vessels:* Vessel segmentation and anatomical labeling are important for vascular disease analysis. The direct use of CNNs for segmentation of vessels in 3D images encounters great challenges. Specifically, head and neck vessels have long and tortuous tubular-like vascular structures with different sizes and shapes. Therefore, it is challenging to automatically and accurately segment and label vessels to expedite vessel quantification. Point cloud representations of head and neck vessels enables quantification of spatial relationships among vascular points. Yao et al. [149] proposed a GCN-based point cloud learning framework to label head and neck vessels and improve CNN-based vessel segmentation on CTA images. To refine vessel segmentation, a point cloud network is first incorporated to the points formed by initial vessel voxels. Then, a GCN is adopted on the point cloud to leverage the anatomical shapes and vascular structures to label the vessel into 13 major segments.

#### 3.4.2. Organ Segmentation

*Cerebral cortex:* The cerebral cortex is the outermost layer of the brain, and is the most prominent visible feature of the human brain. Different regions of the cortex are involved in complex cognitive processes. Reconstructions of the cortical surface captured with sMRI are used to analyse healthy brain organization as well as abnormalities in neurological and neuropsychiatric conditions. Separating the cerebral cortex into anatomically distinct regions based on structure or function is known as parcellation. Traditional CNN approaches have dealt with the mesh segmentation problem by using irregular data represented using graph or mesh structures.

Cucurull et al. [157] investigated the usefulness of graph networks in which contextual information can be exploited for cortical mesh segmentation using the Human Connectome Project data [76,158] (i.e., functional and structural features from cortical surface patches are used for segmentation). The model receives a mesh as input and produces one output label for each node of the mesh, and parcellates the cerebral cortex into three parcels using a graph attention-based model (GAT) [35]. However, brain meshes are constrained within a particular graph structure, ignoring the complex geometry of the surface and hinder all meshes to use the same mesh geometry. Furthermore, the authors conducted cortical parcellation on only selected regions due to memory capacity.

Gopinath et al. [155] leveraged recent advances in spectral graph matching to transfer surface data across aligned spectral domains, and to learn a node-wise prediction. Authors proposed better capabilities for full cortical parcellation on adult brains with GCNs on the MindBoggle dataset [153]. The authors also extend this previous work and proposed a method that learns an intrinsic aggregation of graph nodes based on graph spectral embeddings for cortical region size regression [20].

**Figure 10:**
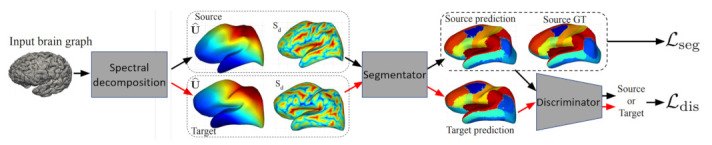
Adversarial graph domain adaptation for segmentation. A cortical brain graph is mapped to a spectral domain. The source and target domain are aligned to a reference template. A GCN segmentator learns to predict a generic cortical parcel label for each domain. Finally, the discriminator classifies the segmentator predictions. Image adapted from [23].

Despite offering more flexibility to analyse unordered data, GCNs are also domain-dependent and are limited to generalize to new domains (datasets) without explicit re-training. Spectral GCNs cannot be used to compare multiple graphs directly and need an explicit alignment of graph eigenbases as an additional pre-processing step. Thus, Gopinath et al. [23] proposed an adversarial graph domain adaptation method for surface segmentation. This approach focused on generalizing parcellation across multiple brain surface domains by eliminating the dependency on domain-specific alignment. In this approach, two networks are trained in an adversarial manner, a fully-convolutional GCN segmentator and a GCN domain discriminator. These networks operate on the spectral components of surface graphs as illustrated in Figure 10. The authors also demonstrate that the model could be useful for semi-supervised surface segmentation, by that alleviating the need for large numbers of labeled surfaces.

**Figure 11:**
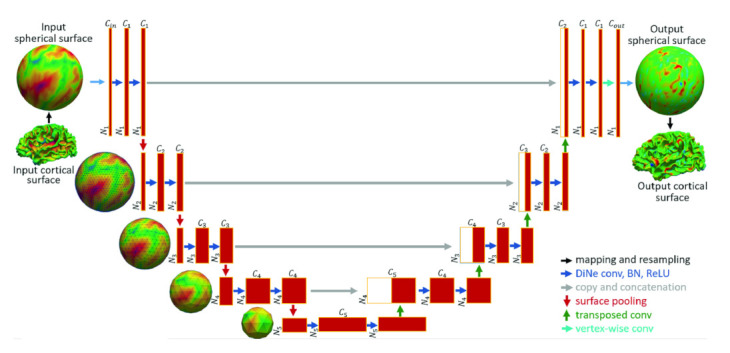
Spherical UNet architecture. The output surface is a cortical parcellation map or a cortical attribute map, and the blue boxes reflect feature maps in spherical space. Note that all spherical surfaces in this figure have the same real size. Image adapted from [19].

Zhao et al. [19] suggested a convolution filter on a sphere, termed Direct Neighbor, which is used to develop surface convolution, pooling and transposed convolution in spherical space. The authors extend the UNet architecture to spherical surface domains as illustrated in Figure 11. The spherical UNet is efficient in learning useful features to predict cortical surface parcellation and cortical attribute map development. Although the method does not rely on spherical registration, it still needs to map cortical surfaces onto a sphere. Spherical mapping is susceptible to topological noise and cortical surfaces are required to be topologically correct before mapping. Therefore, Wu et al. [21] proposed to parcellate the cerebral cortex on the original cortical surface manifold without the need for spherical mapping by taking advantage of the high learning potential of GCNs (i.e., the model is free of spherical mapping and registration). The GCN receives intrinsic patches from the original cortical surface manifold that are mapped using the intrinsic local coordinate system. The extracted intrinsic patches are then combined with the trained models to predict parcellation labels.

Spectral graph matching has been used to transfer surface data across aligned spectral domains, enabling the learning of spectral GCNs across multiple surface data. However, this involves an explicit computation of a transformation map for each brain towards one reference template. He et al. [154] introduced a spectral graph transformer (SGT) network to learn this transformation function across multiple brain surfaces directly in the spectral domain, mapping input spectral coordinates to a reference set. The spectral decomposition of a brain graph is randomly sub-sampled as an input point cloud to a SGT network. The SGT learns the transformation parameters aligning the eigenvectors of multiple brains. The learnt transformation matrix is multiplied by the original spectral coordinates and fed to the GCN for parcellation.

While Laplacian-based graph convolutions are more efficient than spherical convolutions, they are not exactly equivariant. Graph-based spherical CNNs strike an interesting balance, with a controllable trade-off between cost and equivariance (which is linked to performance) [181]. Parvathaneni et al. [156] adopted a deep spherical UNet [182] to encode a relatively large surface mesh. Using a spherical surface registration process, the authors computed deformation fields to produce deformed geometric features that best match ground-truth parcel boundaries. The same authors also implement a spherical UNet for cortical sulci labeling from relatively few samples in a developmental cohort [22]. To enhance the capability of the spherical UNet with limited samples, the authors augmented the geometric features from the training data with their deformed features guided by the intermediate deformation fields. In another work [150], the authors proposed a context-aware training and co-registered every possible pair of training samples for the automated labeling of sulci in the lateral prefrontal cortex in pediatric and adult cohorts.

**Figure 12:**
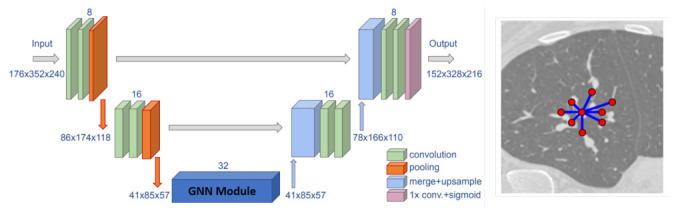
Schematic of a UNet-GNN and illustration of irregular node connectivity for a given voxel in the initial graph. Image adapted from [41].

*Pulmonary airway:* The segmentation of tree-like structures such as the airways from chest CT images is a complex task, with branches of varying sizes and different orientations. Quantifying morphological changes in the chest can indicate the presence and stage of related diseases (e.g., bronchial stenosis). Unlike spheroid-like organs such as liver and kidney, tree-like airways are divergent, thin and tenuous. GNNs were investigated as a way to integrate neighbourhood information in feature utilization for mapping airwaves in the lungs [8,41]. Juarez et al. [41] explored the application of GCNs to improve the segmentation of tubular structures like airways. The authors designed a UNet-GNN architecture by replacing the convolutional layers at the deepest level of the 3D-UNet with a GCN module. The GNN module uses a graph structure obtained from the dense feature maps resulting from the contracting path of the UNet. The GNN learns variations of the input feature maps based on the graph topology, and then outputs a new graph with the same nodes as the input graph, as well as a vector of learnt features for each node. These output feature maps are fed to the up-sampling path of the UNet as illustrated in Figure 12. By introducing a GCN, the method is able to learn and combine information from a larger region of CT chest scans, and is evaluated on the Danish Lung Cancer Screening trial dataset [159].

Selvan et al. [8] also used this volumetric dataset to explore the extraction of tree-structures with a focus on airway extraction, formulated as a graph refinement task, extending the authors’ own prior work [160]. The input image data are first processed to create a graph-like representation, which consists of nodes containing information derived from local image neighbourhoods. Then, a GCN predicts the refined subgraph that corresponds to the structure of interest in a supervised setting, where edge probabilities are predicted from learnt edge embeddings. However, the proposed work treats graph structure learning to be an expensive approximation of a combinatorial optimization problem.

**Figure 13:**
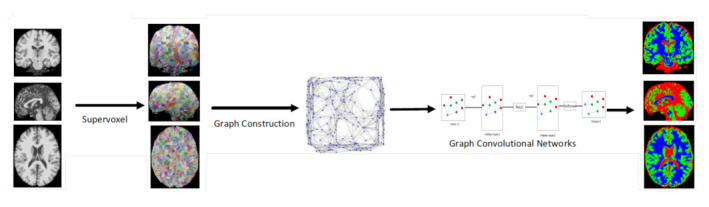
Supervoxels are generated from the brain MRI volume. A graph is constructed from these supervoxels with KNNs. A GCN is employed to classify supervoxels into different types of tissue. Image adapted from [161].

*Brain tissues:* In brain MRI analysis, image segmentation is used for analyzing brain changes, for measuring a brain’s anatomical structures, for delineating pathological regions, and for surgical planning and image-guided interventions. In MRIs of low contrast and resolution, volume effects appear where individual voxels contain different tissues which makes brain tissue segmentation challenging. From several methods in the literature, voxel-wise MRI image segmentation approaches neglect the spatial information within data. As brain MRIs consist of approximately piecewise constant regions, they are well suited to supervoxel generation which has been increasingly used for high dimensional 3D brain MRI volumes. Yan et al. [161] proposed a segmentation model based on GCNs. First, supervoxels from the brain MRI volume are generated; then, a graph is developed from these supervoxels with the *k*-nearest neighbour algorithm used to identify the nodes. Finally, a GCN is adopted to classify supervoxels into different types of tissue such as cerebrospinal fluid, grey matter, and white matter. This framework is illustrated in Figure 13.

*Optic disc/cup and fetal head:* The size of the optic disc and optic cup in color fundus images is also of great importance for the diagnosis of glaucoma, an irreversible eye disease. Meng et al. [164] developed a multi-level aggregation network to regress the coordinates of the boundary of instances instead of using a pixel-wise dense prediction. This model combines a CNN with an attention refine module and a GCN. The attention module works as a filter between the CNN encoder and the GCN decoder to extract more effective semantic and spatial features. Compared to a previous work from the same authors [165], this model also extracts feature correlations among different layers in the GCN. Meng et al. [164] also demonstrated the effectiveness of the network in the segmentation of the fetal head in ultrasound images. Fetal head circumference in ultrasound images is a critical indicator for prenatal diagnosis and can be used to estimate the gestational age and to monitor the growth of the fetus [171]. In this application, the feature map and vertex map size will be different because of different input sizes and the number of contours of instances in the HC18-Challenge dataset [171].

*Pancreas and spleen:* Organ segmentation in CT volumes is an important pre-processing phase for assisted intervention and diagnosis. However, the limitations of expert example annotation and the inter-patient variability of anatomical structures may lead to potential errors in the model prediction. The incorporation of a post-processing refinement phase is a traditional approach to improve the segmentation results. This additional knowledge about the accuracy of the prediction may be helpful in the process. Related to this idea, CNN uncertainty estimation has been used as an attention mechanism for finding potentially misclassified regions for segmentation refinement [183]. Soberanis-Mukul et al. [172,173] proposed a semi-supervised graph learning problem operating over CT data for pancreas and spleen segmentation refinement task. First, a Monte Carlo dropout process is applied to a CNN (2D UNet) to extract the model’s expectation and uncertainty which are used to divide the CNN output into high confidence points (i.e., to find incorrectly estimated elements). This process is represented by a binary mask indicating voxels with high uncertainty. Then, these confidence predictions are used to train a GCN in a semi-supervised way with partially-labeled nodes to refine (reclassify) the output of the CNN. The authors investigated various connectivity and weighting mechanisms to construct the graph. A sparse representation is established that takes into account local and long-range relations between high and low uncertainty elements. Gaussian kernels are used to define the weights for the edges considering the intensity and the 3D position associated with the node. Results on pancreas [174] and MSD-spleen [175] datasets show better performance over traditional CNN prediction with conditional random field refinement.

*Prostate cancer:* MRI is being increasingly used for prostate cancer diagnosis and treatment planning. Accurate segmentation of the prostate has several applications in the management of this disease. However, it is difficult to develop a fully automatic prostate segmentation method that can address various issues, such as variations of shape and appearance patterns in basal and apical regions. Tian et al. [25] proposed an interactive GCN-based prostate segmentation method for MRI. The method is similar to Curve-GCN [184] and adopts a GCN to obtain the coordinates of the contour vertices by regression. The graph module takes the output feature from the CNN encoder applied to the cropped image as its input. Then, the coordinates of a fixed number of vertices from the initial contour are adjusted to fit the target. The interactive GCN model improves the accuracy by correcting the points on the prostate contour with user interactions. Finally, neighbouring points/vertices are connected with spline curves to form the prostate contour. The model outperforms several state-of-the-art segmentation methods on the PROMISE12 dataset [176].

**Figure 14:**
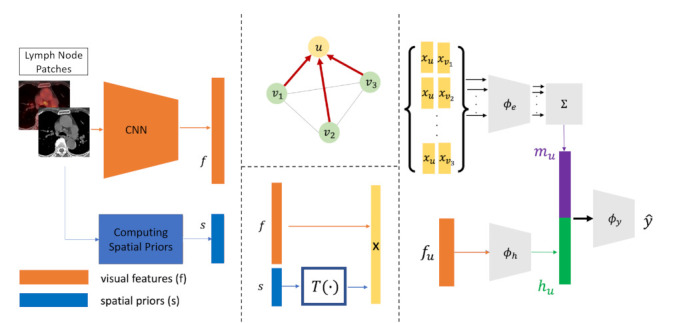
To construct the node representation, the model extracts CNN appearance features and spatial priors for each candidate. Each GTV candidate corresponds to a node in the graph and the GCN is used to exchange information. Image adapted from [178].

*Lymph node gross tumor:* Gross tumor volume (GTV) delineations are a critical step in cancer radiotheraphy planning. In cancer treatment, all metastasis-suspicious lymph nodes (LN) are also required to be treated, which is referred to as lymph node gross tumor volume. The identification of the small and scattered metastasis LNs is especially challenging in non-contrast RTCT. Chao et al. [178] combined two networks, a 3D-CNN and a GNN, to model instance-wise appearance and the inter-lymph node relationships, respectively. Figure 14 depicts this framework. The GNN also models the partial priors computed as the 3D distances and angles for each GTV with respect to the primary tumor. PET imaging is included as an additional input in the CNN to provide complementary information. The model delineates the location of esophageal cancer on an esophageal radiotherapy dataset, outperforming traditional CNN models.

## 4. Research Challenges and Future Directions

Deep learning techniques such as CNN and RNN-based models have demonstrated success in supporting a wide variety of prediction problems in healthcare. However, they are inefficient when dealing with non-Euclidean data representations and when modelling global contextual information. Graph representation learning provides a research avenue to deal with these limitations, by representing such an irregular domain in a meaningful manner, and encoding entity level interactions. As demonstrated throughout this review, graph-based deep learning has attracted significant attention for the analysis of medical data through graph representations of functional connectivity, anatomical structures and electrical activity. However, there are challenges associated with their adoption in this domain that merit further discussion. Advances addressing these challenges would permit GNNs to be extended to a broader variety of domains and applications in circumstances where traditional 2D grid representations are limited. We discuss *five major challenges* that need to be addressed to unlock the full power of graph deep learning: (1) Graph representation; (2) Dynamicity and temporal graphs; (3) Training paradigms and complexity of graph models; (4) Generalization of graph models and deployment; (5) Explainability and interpretability.

At the end of this section, we suggest a new application domain, human behaviour analysis in the medical domain, to which GCNs have not yet been considered, but have great potential to improve patient outcomes.

### 4.1. Graph Representation

Graph neural networks have been used to directly model graph representations of electrical physiological data and medical images including *f*MRI, EEG, MRI and CT data. Defining the graph connectivity structure for a given task is an ongoing problem and in the majority of proposals discussed in this survey, the graph structure is manually designed [67,72]. As there is no standard method for constructing graphs for a GNN model, some authors have used, for example, a bootstrapped version of GCNs that made models less sensitive to the initialisation of the graph structure [69].

Models that infer graph topology from data would be particularly useful when representing diverse types of medical signals with several possible nodes and edges. For ASD analysis, Rakhimberdina et al. [64] used a method to analyse different sets of configurations to build a set of graphs and selected the best performing graph. Jang et al. [81] proposed a model that can automatically extract a multi-layer graph structure and feature representation directly from raw EEG data for affective mental states analysis. However, some essential requirements are still needed to improve the generation process as follows:

*Dynamic weights and node connectivity:* The adjacency matrix should be dynamically learned instead of using predetermined connectivity. During the training process, the weight matrices learn the dynamic latent graph structure. Such approaches have been proposed for MDD [60], ASD [36] and emotion recognition [6,185]. Furthermore, the model should learn both local and global spatial information; however,, in the majority of surveyed works, each node is traditionally connected only to its spatially closest neighbours, which leads to a very limited information exchange between distant nodes.

*Edge attributes:* Edge embeddings in a graph are a poorly studied field with only a few approaches. Learning is principally conducted on the vertices, where the edge attributes supplement the learning as auxiliary information. Edge-weighted models have been studied for the purpose of ASD [17] and BD [44] analysis.

*Embedding knowledge:* Medical domain knowledge can be exploited to solve specific problems by creating networks that seek to mimic the way medical doctors analyse medical data [186]. Graph based mapping with label representations (word embeddings) that guide the information propagation among nodes has been explored [187]. As a result, graph-based analysis motivates researchers to investigate the incorporation of task-specific prior knowledge (e.g., disease label embeddings) in the construction of graph representations such as in the case of chest pathology analysis [114,117].

Building graph generation models using neural networks has also attracted increasing attention to model graphs with complicated topologies and constrained structural properties (e.g., GraphGAN [188]). By modeling graph generation as a sequential process, the model can compute complex dependencies between generated edges. Several of these scalable auto-regressive frameworks that deserve exploration within the medical domain are GraphRNN [189] and GRANs [190].

The above discussion has exemplified the challenges of estimating a graph structure from data with the desired characteristics. While there is work in this field, it is ripe for further exploration. Automated graph generation where a graph model infers the structural content from data are also less explored in the clinical domain.

### 4.2. Dynamicity and Temporal Graphs

Many real-world medical applications are dynamic in nature. In a graph context, this means that a graph’s nodes, edges, and features can change over time. Thus, static embeddings work poorly in temporal scenarios. Several methods that analyse rs-*f*MRI or EEG data discard the temporal dynamics of brain activity, or overlook the functional dependencies between different brain regions in a network. These studies implicitly assume that the brain functional connectivity network is temporally stationary during the entire scanning period. To address this limitation, some works have adopted generic temporal graph frameworks (RNN-based or CNN-based approaches). Such spatio-temporal GCNs that exploit time-varying dynamic information have been demonstrated to outperform traditional GCNs for AD [53] and MDD [60] classification.

Although the dynamicity of graphs can be partly addressed by ST-GCNs, few clinical applications consider how to perform graph convolutions when dynamic spatial relations are present (i.e., nodes, connections or attributes are altered). The majority of spatio-temporal methods in this survey use a predefined graph structure which assumes that the graph has fixed relationships among nodes. Graph WaveNet [58] proposes a self-adaptive adjacency matrix to learn latent static graph structures automatically from data, and the model performs well without being given an adjacency matrix by using a complex CNN-based ST-GNN. Nevertheless, learning latent dynamic spatial dependencies may further improve model accuracy. One method that deserves attention in this direction is ASTGCN [46] which includes a spatial attention function and a temporal attention function to learn latent dynamic spatial and temporal dependencies.

In this challenge, we consider temporal graphs and the need for dynamic graphs which can be applied to data where dependencies and the underlying structure changes over time. This challenge is tightly coupled to the previous challenge of learning graph representations but adds the additional complication of seeking to learn how connections change over time. Apart from the work proposed for sleep stage classification [45], the research on adaptive graphs for spatio-temporal analysis is limited.

### 4.3. Training Paradigms and Complexity of Graph Models

Training a GNN remains difficult due to their high memory consumption and inference latency. However, the adoption of efficient training approaches is uncommon in the applications surveyed. Various graph sampling approaches have been proposed as a way to alleviate the cost of training GNNs such as GraphSage [30], which proposes a batch-training algorithm to save memory. Other potential methods of improvement in speed and optimization include PinSage [191], fast learning with GCN (FastGCN) [192], stochastic GCNs (StoGCN) [193], Cluster-GCN [194] and layer-wise GCN (L-GCN) [195]. Hence, further investigation should be carried out to introduce such strategies into the medical domain.

To learn rich representations, the majority of approaches discussed in this survey require task-dependent labels which is a critical challenge for medical applications that rely on supervised training. As such, it could be extremely beneficial if the training mechanism can extract information from unlabeled samples. Although the issue of scarce or missing annotations is a general problem not specific to the graph domain, only a few works have adopted viable solutions to address this limitation by following research directions such as weakly supervised learning and semi-supervised learning. Weakly-supervised and semi-supervised algorithms with graph models have been widely explored in computational histopathology [26], but further research is required to investigate such methods for use with electrical activity data and anatomical structures. Only a few works have used semi-supervised learning for the segmentation of the cerebral cortex [23] and organs [172].

Self-supervised learning is another research direction with significant potential. When no class labels are available in the graph, graph embeddings can be learnt using an end-to-end method in an entirely unsupervised manner. Although self-supervision in graphs [196] and graph convolutional adversarial networks for unsupervised domain adaptation [197] have been investigated to improve the performance of models, we observe that their adoption has not yet emerged in the medical domain works surveyed. Therefore, further investigation can be carried out to assess the viability of such techniques. One method is to use an autoencoder framework, in which the encoder uses graph convolutional layers to embed the graph in a latent representation, which is then reconstructed using a decoder [4]. Recent works on contrastive learning by optimizing mutual information between node and graph representations [198] have achieved state-of-the-art results on both node classification [199] and graph classification tasks [200].

One aspect of graph-based deep learning not yet discussed for medical applications is reinforcement learning (RL), which learns from experiences by interacting with the environment. RL can address the limitations of supervised learning with robust and intuitive algorithms trainable on small datasets [201]. There are opportunities to employ RL in multi-task and multi-agent learning paradigms where graph convolutions adapt to the dynamics of the underlying graph of the multi-agent environment [202], or to apply RL for graph classification using structural attention to actively select informative regions in the graph [203].

Another limitation that we observe in present research is that GCNs share considerable computational complexity from their deep learning lineage which can be burdensome for scaling and deploying GNNs. Some experiments have shown that the performance of GCNs drops dramatically with an increase in the number of graph convolutional layers [204]. This raises the question of whether going deeper is still a good strategy for learning from graph data [205]. Therefore, the choice of GNN architecture should be treated as a hyperparameter in the proposed clinical application.

Efficient and simple architectures have been also proposed to reduce the complexity of GCNs. One example is the simple graph convolution (SGC) network [39] which reduces complexity by collapsing multiple weight matrices into a single linear transformation, and eliminating nonlinearities between GCN layers. This model was adopted for the evaluation of ASD and ADHD [40]. Other approaches that merit consideration are the simple scalable inception GNN (SIGN) [206] and the efficient graph convolution (EGC) [207].

There has been a growing interest in the literature in graph embedding problems, but these approaches do not usually scale well to real-world graphs. Recent advances in distributed and batch training for graph neural networks look promising but they require hours of CPU training, even for small and medium sized graphs. The use of accelerators such as GPU-based tools to deal with graphs is largely under-explored [208]. To address this, Akyildiz et al. [209] introduced GOSH, which utilizes a graph partitioning and coarsening approach, a process in which a graph is compressed into smaller graphs, to provide fast embedding computation on a single GPU with minimal constraints.

How to effectively compute GNNs in order to realise their full potential will be a key research topic in the coming years. Several hardware accelerators have been developed to cope with the high density of GNNs and alternating computing requirements, but there is not a clear solution applicable to multiple GNN variants [210]. On the software side, current deep learning frameworks including extensions of popular libraries such as TensorFlow and PyTorch have limitations when implementing dynamic computation graphs along with specialized tensor operations [210]. Thus, there is a need to further develop libraries such as, for example, DGL [211], which may handle the sparsity of GNN operations efficiently, as well as complex tensor operations in CUDA with GPU computation acceleration.

### 4.4. Generalization of Graph Models and Deployment

Several graph methods suffer from challenges posed by inter-site heterogeneity caused by different scanning parameters and protocols, and from subject populations at different sites. It is difficult to build accurate and robust learning models with heterogeneous data. Due to patient privacy and clinical data management requirements, truly centralized open source medical big data corpora for deep learning are rare. Medical applications are hindered by non-generalizability that limits deployment to specific institutions. To alleviate the heterogeneity, simultaneously learning adaptive classifiers and transferable features across multiple sites and subjects offers a promising direction.

Transfer learning provides a potential solution by transferring well-trained networks on large scale datasets (related to the to-be-analyzed disease) to a small sample dataset. Such generalization across datasets was demonstrated by capturing essential dementia-associated patterns from different datasets [104].

Another interesting research direction for investigation is domain adaptation in which the source and target domains have the same feature space but different distributions. This also aims to deal with multiple domains and even multiple heterogeneous tasks [212]. The generalizability of trained classifiers in subject-independent classification settings is hampered by the considerable variation in physiological data across different subjects. Some works have attempted to tackle this challenge by introducing domain adversarial training [213]. Graph adversarial methods adopt adversarial training techniques to enhance the generalization of graph-based models. Such domain adaptation methods have been introduced for cerebral cortex segmentation [23], brain data prediction [7,130] and emotion recognition [214].

Meta-learning, a sub-field of transfer learning, has been used in areas including task-generalization problems such as few-shot learning. Meta-learning is the ability to learn, also known as “learning to learn”. Few-shot learning (FSL) aims to automatically and efficiently solve new tasks with few labeled samples based on knowledge obtained from previous experiences. These models are emerging in the medical domain [215] for decoding brain signals [216] and a few approaches have explored GNNs for few-shot learning [217]. Another interesting variant of transfer learning is zero-shot learning (ZSL), which aims to predict the correct class without being exposed to any instances belonging to that class in the training dataset. Although zero-shot learning is flourishing in the field of computer vision [218], it is seldom used for biomedical signal analysis, though zero-shot learning has recently been used to recognize unknown EEG signals [219]. Recently, GCNs have shown a lot of promise for zero-shot learning. When encountered with a lack of data, these models are highly sample efficient because related concepts in the graph structure share statistical strength, allowing generalization to new classes [220]. Knowledge graphs can also be used to guide zero-shot recognition classification as extra information [220], making these models a notable future research prospect.

We analyze the challenges that affect the ability of GCNs to generalize, including to unknown tasks and domains, and the potential research directions this offers. A number of interesting paths exist, including the development of meta-models improving knowledge generalization, enabling the more rapid deployment of applications.

### 4.5. Explainability and Interpretability

A lack of transparency is identified as one of the main barriers for AI adoption in clinical practice. Physicians are reluctant to trust a machine learning model’s predictions because of a lack of evidence and difficulty in interpreting the reasons for a decision, particularly in disease diagnosis. A step towards trustworthy AI is the development of explainable AI [221]. Explainable AI seeks to create insights into how and why AI models produce predictions. A natural question that arises is if the decision-making process in deep learning models can be interpretable.

Several prominent explainability methods for deep models have been used to provide input-dependent explanations using visual explanations and salient regions. Existing methods include gradient-based methods such as guided backpropagation, or class activation maps (CAM), and the generalized versions Grad-CAM and Grad-CAM++ [222]. While these methods have been explored in a number of areas, they were not proposed to address interpretability in a clinical context. Clinical areas bring additional requirements such as the incorporation of the physician’s interpretation (i.e., to satisfy a meaningful explanation, the model should “explain” its output in such a way that physicians can understand). Interpretability for graph-based deep learning is significantly more challenging than for CNN or RNN-based models because graph nodes and edges are often heavily interconnected. The main challenge of redesigning and applying existing CNN explainers to GNNs is that they fail to incorporate relationship information, which results in ill-defined visual heatmaps of important regions [223].

Model-based and post-hoc interpretability are the two most common types of interpretation approaches. The former constrains the model so that it readily provides useful details about the uncovered relationships (such as sparsity, modularity, etc). The latter attempts to extract information about the model’s learned relationships [224,225]. These post-hoc methods are typically used to analyze individual feature input and output pairs, limiting their explainability to an individual-level. Several explanation methods have been presented in the literature including layer-wise relevance propagation (LRP) [225], excitation backpropagation [224], graph pruning (GNNExplainer) [223], gradient-based saliency (GraphGrad-CAM) [226], GraphGrad-CAM++ [227]), and layerwise relevance propagation (GraphLRP) [228].

While there is much interesting research within the field of interpretability for GNNs, it is immature and there are only a few papers that explore these methods across the ones surveyed. Post-hoc explanation techniques have been used to visualize attention weights or to recognize relevant subgraphs for classification. Such explanation techniques have been useful, for example, to identify brain regions that are most involved when a seizure occurs [59,86], and key brain regions associated with biomarkers for PD [47] or ASD [16,17,61] prediction. The activation map and gradient sensitivity of graph models are also used to interpret the salient input features at both the group and individual levels for the analysis of BD [44] and ASD [61]. Both individual-level and group-level explanations are critical in medical research. Individual-level biomarkers are desirable for planning targeted care in precision medicine, while group-level biomarkers are essential for understanding disease-specific characteristic patterns. Although attention weights for edges can be used to measure edge importance, it is noted that they can only explain GAT models without explaining node features, unlike, for example, GNNExplainer [223].

We believe further extensive investigation of traditional gradient-based (GraphGrad-CAM) and perturbation-based methods (GNNExplainer) for instance-level interpretation in the medical domain will allow rapid explainability at the node, edge, or node feature levels. Other methods that could be extensively utilised within this research direction are PGM-explainer [229] and SubgraphX [230]. Studies examining the effect of explanations on clinical end-user decisions show generally positive results. Thus, further studies should also investigate how to integrate the clinical workflow into the model, and how a clinical expert could refine a model decision via a human-in-the-loop process. One such example introduced an interactive GCN-based prostate segmentation method [25] where the annotator can choose any wrong control points and correct these via user interactions.

The implementation of deep-learning based models raises complex clinical and ethical challenges due to difficulties in understanding the logic involved in these models. Interpretability is essential as it can help informed decision-making during diagnosis and treatment planning. However, GCNs are complex models and interpreting the model’s outcome remains a challenging task. Interpretability techniques are gaining importance in recent years. However, aside from the study in computational pathology [26] which is outside the scope of this review, the interpretability of graph neural networks in a clinical context has not been addressed sufficiently. Considering the spread of graph-based processing for various medical applications, graph explainability and its quantitative evaluation with a focus on usability by clinicians are crucial.

### 4.6. Future Prospects of Graph Neural Networks for Patient Behavioural Analysis

Medical applications have benefited from rapid progress in the field of computer vision. Up to this point, the majority of studies have concerned themselves with analysing data that results from diagnostic procedures, and using this to predict the presence of a disease. As a result of this focus, the areas of patient behaviour assessment have received less attention. While several in-clinic systems using CNN and RNN-based models have been introduced to enable comprehensive data analysis through accurate and granular quantification of a patient’s movements [231,232,233], these methods are not yet sufficiently accurate for widespread clinical use, yet we argue that graph neural networks have a great potential in these application areas.

Image classification, regression and segmentation have been addressed with CNN models by excelling at modeling local relations. However, GCNs can take into account different neighbouring relations (global relation) by going beyond the local pixel neighbourhoods used by convolutions. Graph embeddings have appeared in other computer vision tasks where relations between objects can be efficiently described by graphs, or for the purpose of graph-structured image data analysis [4,12]. The adoption of graph-based deep learning models have also been explored for the analysis of human actions. Below, we highlight research directions based on graph models to support the highly relevant clinical domain of behavior monitoring, and motor and mental disorder assessment tools.

- Facial analysis: Clinical experts rely on certain facial modifications and symptoms for assistive medical diagnosis, and computer vision has been introduced to offer an automatic and objective assessment of facial features. Interesting results have been obtained by incorporating graph-based models for facial expression recognition [234], action unit detection [235] and micro-expression recognition [236].
  *Potential applications*: Postoperative pain management, monitoring vascular pulse, facial paralysis assessment, and several neurological and psychiatric disorders including seizure semiology, ADHD, autism, bipolarity and schizophrenia.
- Human pose localization: Since human pose estimation is related to graph structure, it is important to design appropriate models to estimate joints that are ambiguous or occluded. GCNs can process skeleton data in a flexible way to improve the skeleton structure’s expressive power. GCNs have been used to refine 2D human pose localization [237], 3D human pose estimation [238], and multi-person pose estimation [239].
  *Potential applications*: In-bed pose estimation to track pressure injuries from surgery and illness recovery, and other sleep disorders such as apnea, pressure ulcers, and carpal tunnel syndrome.
- Pose-based action recognition: Movement assessment and monitoring is a powerful tool during clinical observations where uncontrolled motions can aggravate wounds and injuries, or aid the diagnosis of motor and mental disorders. These motions are represented as continuous time-series of the kinematics of the head, limbs and trunk movements. Given a time-series of human joint locations, GCNs have been widely used to estimate human action patterns [56,240,241,242]
  *Potential applications*: Motor disorders (Epilepsy, Parkinson’s, Alzheimer’s, stroke, tremor, Huntington and neurodevelopmental disorders); mental disorders (Dementia, schizophrenia, major depressive, bipolar and autism spectrum); and other situations including breathing disorders, inpatient fall prediction, and health conditions such as agitation, depression, delirium, and unusual activity.

Graph representations for skeleton-based action recognition are gaining prominence over the last couple of years. Apart from initial studies to flag abnormal behaviour in dementia [243], assessment of Parkinsonian leg agility and gait [244,245], and human emotion based on skeleton detection [246], graph neural networks in the context of in-bed pose estimation and patient behaviour estimation are poorly investigated compared to other computer science fields.

## 5. Conclusions

Functional, anatomical and electrical data provide essential information on many diseases’ etiology, onset, and progression, as well as treatment efficacy. Our survey provides a comprehensive review of research on graph neural networks and their application to medical domains and applications including functional connectivity, electrical, and anatomical analysis. Digital pathology has not been the main focus of this survey, and we have sparsely mentioned the applications of GCNs to this domain. However, considering the comprehensive application of deep learning to digital pathology (WSI), readers are referred to a complementary survey that thoroughly covers the potential applications of GCN to WSI [26].

As we have shown in this review, the growing mass of literature in this space and the rapid development and search for new tools and methods suggest that we are at the verge of a paradigm shift. Furthermore, considering the remarkable ability of GCNs in dealing with unordered and irregular data such as brain signals, and their simplicity and scalability, graph-based deep learning will progressively take a more prominent role and complement traditional machine learning approaches.

Recent advances in the adoption of graph-based deep learning models for classification, regression and segmentation of medical data show great promise. However, we have outlined several challenges related to their adoption, including the graph representation and estimation, graph complexity, dynamicity, interpretability and generalization of graphs. These and many other challenges lead to a vast amount of open research directions, the solutions to which will benefit the field and lead to many applications in the medical domain. This constitutes a clear challenge to the neuroengineering scientific community, and it is hoped that the community will increase their efforts to address these emerging challenges. Although one will never replace the power of individual clinical expertise, by providing more quantitative evidence and appropriate decision support, one can definitely improve medical decisions and ultimately the standard of care provided to patients.

## Data Availability

Not applicable.
